# Incorporation of rapid association/dissociation processes in tissues into the monkey and human physiologically based pharmacokinetic models for manganese

**DOI:** 10.1093/toxsci/kfac123

**Published:** 2022-12-01

**Authors:** Jerry L Campbell, Harvey J Clewell, Cynthia Van Landingham, P Robinan Gentry, Athena M Keene, Michael D Taylor, Melvin E Andersen

**Affiliations:** Ramboll US Corporation, Raleigh, North Carolina, USA; Ramboll US Corporation, Raleigh, North Carolina, USA; Ramboll US Corporation, Monroe, Los Angeles, USA; Ramboll US Corporation, Monroe, Los Angeles, USA; Afton Chemical Corporation, Richmond, Virginia, USA; NiPERA, Durham, North Carolina, USA; Andersen ToxConsulting LLC, Chapel Hill, North Carolina, USA

**Keywords:** manganese, physiologically based pharmacokinetic model, interspecies extrapolation, pharmacokinetics

## Abstract

In earlier physiologically based pharmacokinetic (PBPK) models for manganese (Mn), the kinetics of transport of Mn into and out of tissues were primarily driven by slow rates of association and dissociation of Mn with tissue binding sites. However, Mn is known to show rapidly reversible binding in tissues. An updated Mn model for primates, following similar work with rats, was developed that included rapid association/dissociation processes with tissue Mn-binding sites, accumulation of free Mn in tissues after saturation of these Mn-binding sites and rapid rates of entry into tissues. This alternative structure successfully described Mn kinetics in tissues in monkeys exposed to Mn via various routes including oral, inhalation, and intraperitoneal, subcutaneous, or intravenous injection and whole-body kinetics and tissue levels in humans. An important contribution of this effort is showing that the extension of the rate constants for binding and cellular uptake established in the monkey were also able to describe kinetic data from humans. With a consistent model structure for monkeys and humans, there is less need to rely on cadaver data and whole-body tracer studies alone to calibrate a human model. The increased biological relevance of the Mn model structure and parameters provides greater confidence in applying the Mn PBPK models to risk assessment. This model is also well-suited to explicitly incorporate emerging information on the role of transporters in tissue disposition, intestinal uptake, and hepatobiliary excretion of Mn.

Manganese (Mn) is an essential trace element found in all human tissues and is required for numerous physiological processes, including protein and carbohydrate metabolism, immune system function, and bone growth. When Mn intake exceeds elimination, Mn can accumulate in mid-brain regions that influence motor control, such as the globus pallidus, striatum, and substantia nigra (Dorman *et al.*, 2006b; Yamada *et al.*, 1986), resulting in neurotoxicity. Similar neurological responses have been linked to prolonged inhalation exposures (Pal *et al.*, 1999) or ingestion of drinking water with high concentrations of Mn (Kawamura, 1941), or in patients with liver disease resulting in impaired Mn clearance (Spahr *et al.*, 1996), and with long-term parenteral nutrition (Fell *et al.*, 1996). For human risk assessment, it is important to determine the exposure conditions that result in Mn concentrations in the brain that are increased significantly compared with brain Mn concentrations arising from normal dietary intake (Andersen *et al.*, 1999).

Concerns surrounding chronic low-level Mn inhalation exposure have led to the development of an extensive Mn pharmacokinetic data set and physiologically based pharmacokinetic (PBPK) models for rats, monkeys, and humans (Dorman *et al.*, 2012; Taylor *et al.*, 2012). A series of pharmacokinetic approaches have been used to describe Mn kinetics, including compartmental models (Teeguarden *et al.*, 2007a,b,c), PBPK models of adult rats, monkeys, and humans (Nong *et al.*, 2008, 2009; Schroeter *et al.* 2011, 2012; Yoon *et al.* 2019), and PBPK models of gestation and lactation in rats (Yoon *et al.*, 2009a,b). The initial Mn compartmental PK models that relied on linear first-order processes to simulate Mn tissue kinetics under normal and deficient dietary conditions were unable to capture the rapid rise in tissue Mn concentrations seen during inhalation exposure to high Mn concentrations. A revised PBPK model structure was developed in the rat and monkey that allowed tissue compartments to maintain near constant Mn levels during normal dietary intake and included Mn tissue stores that could become saturated at higher exposures (Nong *et al.*, 2009). Schroeter *et al.* (2011) extended the Nong *et al.* (2009) PBPK model for rats and monkeys to humans to predict inhalation exposure conditions expected to result in increased brain Mn concentrations in humans. This modeling effort included simulation of intravenous (iv), intraperitoneal (ip), and subcutaneous (sc) routes. This work also allowed for the analysis of studies of tracer kinetics of ^54^Mn in monkeys and human volunteers as well as bulk Mn in tissues. These tracer studies with soluble carrier-free ^54^Mn (given as ^54^MnCl_2_) reflect the overall kinetics of Mn in the body at different body burdens of Mn.

Yoon *et al.* (2019) recast the PBPK model for the adult rat to have more rapid binding of Mn to and its dissociation from binding sites in tissues and more rapid tissue uptake. This effort was based on detailed understanding of the processes important to Mn homeostasis, including avid, rapidly reversible binding with multiple proteins/enzymes within tissues (Das *et al.*, 2019; Wedler, 1993) and facilitated transport of Mn into and out of tissues via cellular Mn transporters (Aschner and Erikson, 2017; Chen *et al.*, 2015). Several solute transporter proteins are involved in Mn homeostasis. In addition to divalent metal transporter 1 (DMT1), Zip-8 and transferrin/transferrin receptor system, SLC30A10, SLC39A8, and SLC39A14 are key transporters in Mn clearance and maintenance of Mn homeostasis in vertebrates (Chen *et al.*, 2014, 2015; Leyva-Illades *et al.*, 2014; Lin *et al.*, 2017; Mercadante *et al.*, 2019; Tuschl *et al.*, 2016). Compared with Mn importers, an understanding of transporters for Mn efflux and the underlying mechanisms of Mn efflux and their role in maintaining Mn homeostasis in mammalian systems is more recent. SLC30A10 appears to be one of the more relevant Mn efflux transporters and plays a role in mediating Mn efflux in neuronal systems (Chen *et al.*, 2015; Hutchens *et al.*, 2017; Leyva-Illades *et al.*, 2014).

In this study, a model structure with more rapid association-dissociation processes for Mn binding in tissues, first developed for the rat (Yoon *et al.* 2019), was extended to create similar PBPK model structures for monkey and human. These updated PBPK models more accurately represent the current state of the knowledge of Mn biology and will facilitate the incorporation of data from knowledge of Mn stores in tissues and the emerging information from both *in vitro* and *in vivo* studies on Mn transport. This more realistic PBPK model for Mn increases confidence in its use for human risk assessment.

## Materials and methods

###  

####  

##### PBPK model structure

**Figure 1.**
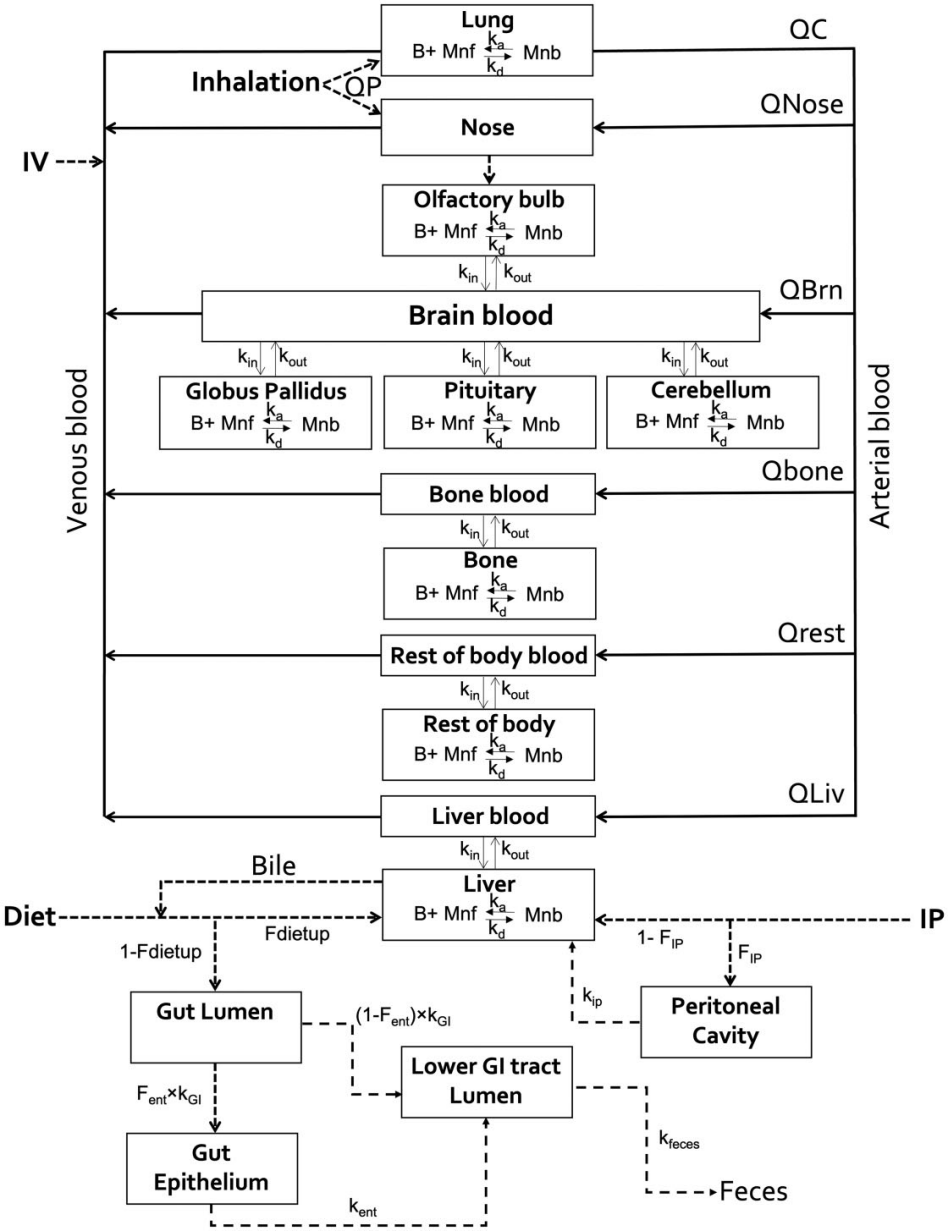
Manganese PBPK model schematic.

This updated Mn PBPK model structure contains compartments for liver, lung, nasal cavity, bone, blood, cerebellum, olfactory bulb, globus pallidus, and pituitary gland (Figure 1). Physiological parameters for the monkey and human are reported in Table 1. An aggregated body tissue compartment represents all other tissues. The same model structure was used to examine data on Mn kinetics derived from studies with both monkeys and humans. The same kinetic data sets that were relied upon for this model had been employed in an earlier model that relied on a model structure with much slower association-dissociation processes (Schroeter *et al.*, 2011, 2012). The manganese model presented here includes an expanded GI absorption description which can be adjusted to account for the dose-dependent uptake of orally administered manganese. This allows for simulation of concurrent exposure to dietary and inhaled Mn, and to simulate ^54^Mn tracer kinetics from oral and inhalation exposure and from ip, iv, and sc dose routes.

**Table 1.** Physiological parameters for monkey and human

Parameter	Monkey	Human	Units
Body weight (kg)	5.0^a^	73 (60)^b^	kg
Tissue volumes (fraction of body weight)			
Blood	0.0734^a^	0.0767 (0.0683)^b^	
Bone	0.144^c^	0.144 (0.130)^b^	
Brain	0.018^a^	0.020 (0.022)^b^	
Cerebellum (fraction of brain)	0.085^d^	0.0831^e^	
Olfactory bulb (fraction of brain)	0.00056^d^	0.000079^f^	
Globus Pallidus (fraction of brain)	0.0022^d^	0.0024^g^	
Pituitary (fraction of brain)	0.00037^d^	0.000414^b^	
Liver	0.030^a^	0.0247 (0.233)^b^	
Lung	0.0066^a^	0.0076 (0.0070)^b^	
Remaining body	BW - Sum of other tissue fractions		
Surface area of nasal respiratory epithelium	13.42^h^	8.25^i^	cm^2^/BW^0.75^
Surface area of nasal olfactory epithelium	1.54^h^	0.95^i^	cm^2^/BW^0.75^
Average nasal tissue thickness	375^j^	375^j^	µm
Cardiac output	19.5^a^	16.5^k^	l/h/kg^0.75^
Pulmonary ventilation	30.0^a^	24.0^k^	l/h/kg^0.75^
Tissue blood flow (fraction of cardiac output)			
Bone	0.042^c^	0.05^b^	
Brain	0.065^a^	0.12^b^	
Liver	0.201^a^	0.255 (0.270)^b^	
Nose	0.01^c^	0.01^l^	
Remaining body	Difference^m^		

a Davies and Morris (1993), study-specific values used where reported.

b ICRP (2002) study-specific values used where reported, male (female where different).

c Set to human value.

d Dorman *et al.* (2006a). Note: monkey cerebellum, olfactory bulb, and globus pallidus were calculated as 2 time’s the right hemisphere volume as reported (see Supplementary Table 1).

e Taman *et al.* (2020) fraction of ICRP 2020 brain volume (1.45 l).

f Poessel *et al.* (2020) fraction of ICRP 2020 brain volume (1.45 l).

g Kijonka *et al.* (2020) fraction of ICRP 2020 brain volume (1.45 l).

h Menache *et al.* (1997), using fraction to total nose for olfactory and respiratory tissue for human repoted by Schroeter *et al.* (2008).

i Schroeter *et al.* (2008); olfactory = 23.4/72^0.75^ and respiratory = 203.9/72^0.75^.

j Conolly *et al.* (2000).

k Clewell *et al.* (2001).

l From Frederick *et al.* (1998).

m Difference 1.0 − (sum of listed compartment).

The inhalation exposure studies in monkeys that were relied upon for kinetic data sets were carried out with MnSO_4_ particles, which are highly soluble in mucus and more rapidly taken up by the respiratory tract (Vitarella *et al.*, 2000). The aerosol parameters for MnSO_4_ consisted of a mass median aerodynamic diameter of 2.0 µm, a geometric standard deviation of 1.5, and a particle density of 2.95 g/cm^3^ (Dorman *et al.*, 2006a). The fractional deposition of inhaled MnSO_4_ particles in the respiratory tract were calculated using the multiple-path particle dosimetry model (MPPD version 3.04; Anjilvel and Asgharian, 1995; Asgharian *et al.*, 2001). Nasal deposition estimates from the MPPD model were partitioned onto the respiratory and olfactory epithelium based on species-dependent airflow allocation (Schroeter *et al.* 2011). Deposited Mn was assumed to be rapidly absorbed from lung tissues and nasal respiratory epithelium into the systemic circulation or transported from the nasal olfactory epithelium to the olfactory bulb.

GI absorption of Mn is dose dependent. GI absorption decreases and biliary excretion increases as dietary Mn levels increase (Dorman *et al.*, 2006b; Teeguarden *et al.*, 2007b). Previously established parameters including the fraction of Mn absorbed by the GI tract (FDIETUP) and the biliary excretion rate constant (KBILEC) were retained from the Schroeter *et al.* (2011) model, which had been calibrated based on steady-state tissue concentrations and ^54^Mn whole-body elimination curves in monkeys. Induction of biliary elimination of Mn, which was included to describe increased bile elimination that was directly observed in higher exposure concentrations in monkeys (Nong *et al.*, 2009), was retained in this effort. The biliary elimination rate was dependent on blood Mn concentration and followed a Michaelis-Menten pattern of induction:

(1) $$\mathrm{KBILEX}=\mathrm{KBILE}*\left(1+\frac{\mathrm{KBINDUC}\;*\;{\mathrm{Cart}}^{n}}{{\mathrm{KM}}^{n\;}+\;{\mathrm{Cart}}^{n}}\right)$$

where KBILEX (l/h) is the rate of biliary excretion of Mn, KBILE is the allometrically scaled basal biliary excretion rate, KBINDUC, is the maximal increase in the biliary excretion rate, Cart is the concentration of Mn in the arterial blood, KM is the arterial concentration leading to half maximal induction, and *n* (parameter label is SLOPE in model file) is a Hill-type induction constant. The arterial blood concentration was used as a surrogate for free Mn liver concentrations, the presumed driver for Mn excretion, because Mn blood levels were directly measured in monkeys (Dorman *et al.*, 2006a; Nong *et al.*, 2009).

We also modified the GI structure reported by Nong *et al.* (2009) to include fecal excretion in order to simulate the tracer kinetics of ^54^Mn administered orally in monkeys and humans. To do this, a multicompartment gut was added to be consistent with the physiology of Mn absorption by the GI tract (Figure 1), following the approach of Schroeter *et al.* (2011). FDIETUP represents the fraction of dietary Mn absorbed from the GI tract and available to the systemic circulation. This process was described as a direct transfer from the gut lumen to the liver. The remaining fraction of Mn (1-FDIETUP) in the gut lumen is either retained in the gut in a gut tissue storage compartment representing enterocytes or directly transported to the lower GI tract lumen from which it is excreted in the feces. *F*_ENT_ * *K*_GI_ represents the storage rate constant into the enterocytes, where *F*_ENT_ is the fraction stored and *K*_GI_ is the rate constant for movement from the gut lumen. Mn transfer from the gut tissue storage compartment to the lower GI tract represents sloughing of the intestinal epithelial cells with a rate constant *K*_ENT_. This sloughed Mn is excreted in the feces without entering the systemic circulation. Mn elimination from the body in the feces was governed by the rate constant *K*_FECES_. The GI parameters reported in Schroeter *et al.* (2011) were retained for this effort.

##### Tissue transport and binding model

**Figure 2.**
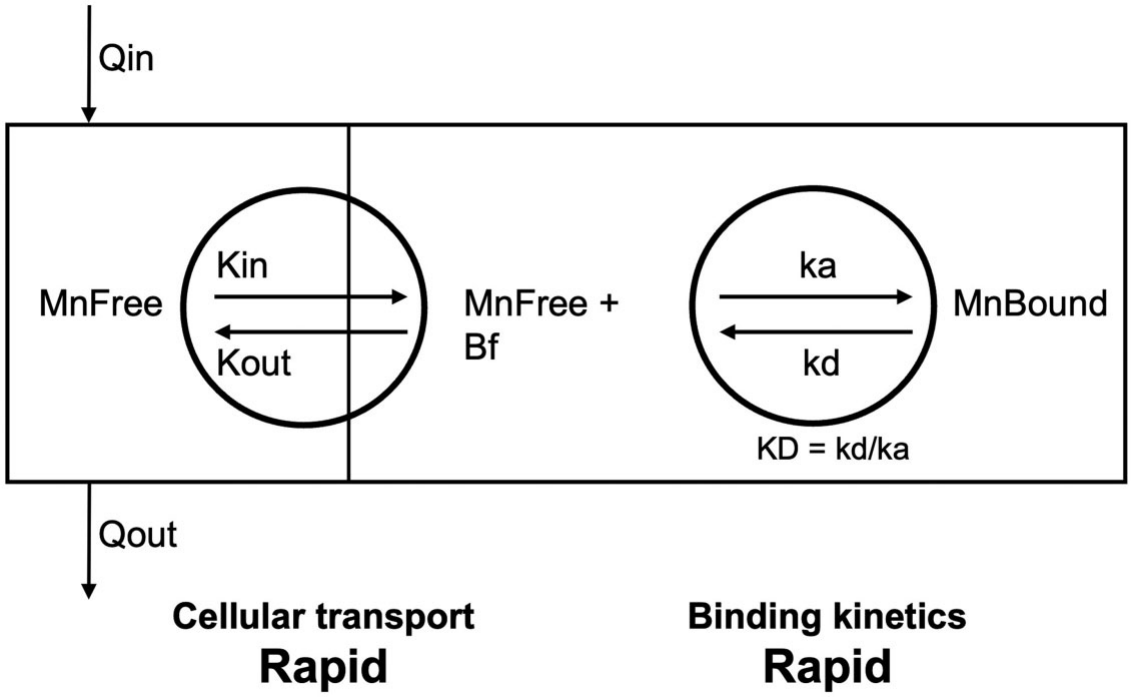
Mn transporter model tissue structure as described by Yoon *et al.* (2019). The *K*_in_ and *K*_out_ represent diffusion rate constants for Mn influx and efflux, respectively, whereas the *k*_A_ and *k*_D_ represent association and dissociation rate constants. Mn total is represented by MnFree plus MnBound, whereas Bmax (maximal binding capacity in a tissue) is represented by Bf (binding sites available for binding) plus MnBound.

Transport and binding processes in Yoon *et al.* (2019) differed from those in the original Mn-PBPK models (Nong *et al.*, 2009; Schroeter *et al.*, 2011, 2012). In the earlier Mn PBPK models, the movement of Mn between the tissue and blood was relatively rapid while the association and dissociation rate of bound forms in tissue were slow and designed to be the limiting process for loss of Mn when body and tissue stores were reduced by alterations in intake or at the end of high dose exposures. In the revised Mn model (Figure 2) developed by Yoon *et al.* (2019), the binding and dissociation rate constants are much larger allowing tissue stores to adjust faster than was possible in the original model. The higher rates of tissue binding and the consistency of the dissociation equilibrium constant across tissues is more reflective of the biology of interactions of Mn with tissue proteins and tissue organelles (Das *et al.*, 2019; Wedler, 1993). The use of a common dissociation constant (*K*_D_ ∼ 0.5 μM) is also more consistent with similarities in Mn utilization and requirements across tissue types leading to the maintenance of Mn bound forms until the binding sites become saturated (Yoon *et al.* 2019). Variability in the presumed binding capacities across tissues still accounts for different background levels of Mn in particular tissues, as was the case in the original Mn model of Nong *et al.* (2008), and accounts for differing Mn-requiring processes in different tissues. For this update to the monkey and human Mn models, the equilibrium binding constant, *K*_D_, of approximately 0.5 μM and the association and dissociation rate constants for all tissues (Table 2) were the same as used by Yoon *et al.* (2019).

**Table 2.** Chemical specific parameters for the monkey and human Mn PBPK model

Label	Description	Monkey	Human	Scaling	Source
Cellular transport					
KINSTC	Striatum influx rate constant	0.092	0.092	(BW^0.25^/h)	Optimized to monkey
KINCBC	Cerebellum influx rate constant	0.74	0.74	(BW^0.25^/h)	Optimized to monkey
KINOBC	Olfactory bulb influx rate	2.04	2.04	(BW^0.25^/h)	Optimized to monkey
KINPTC	Pituitary influx rate constant	0.016	0.016	(BW^0.25^/h)	Optimized to monkey
KINLUNGC	Lung influx rate constant	6.61	6.61	(BW^0.25^/h)	Optimized to monkey
KINLIVC	Liver influx rate constant	127.94	127.94	(BW^0.25^/h)	Optimized to monkey
KINBONEC	Bone influx rate constant	37.58	37.58	(BW^0.25^/h)	Optimized to monkey
KINOTHC	Rest of body (other) influx rate constant	0.76	0.76	(BW^0.25^/h)	Optimized to monkey
KOUTSTC	Striatum efflux rate constant	0.027	0.027	(BW^0.25^/h)	Optimized to monkey
KOUTCBC	Cerebellum efflux rate constant	0.022	0.022	(BW^0.25^/h)	Optimized to monkey
KOUTOBC	Olfactory bulb efflux rate constant	16.69	16.69	(BW^0.25^/h)	Optimized to monkey
KOUTPTC	Pituitary efflux rate constant	0.012	0.012	(BW^0.25^/h)	Optimized to monkey
KOUTLUNGC	Lung efflux rate constant	0.071	0.071	(BW^0.25^/h)	Optimized to monkey
KOUTLIVC	Liver efflux rate constant	2.86	2.86	(BW^0.25^/h)	Optimized to monkey
KOUTBONEC	Bone efflux rate constant	0.51	0.51	(BW^0.25^/h)	Optimized to monkey
KOUTOTHC	Rest of body (other) efflux rate constant	0.0049	0.0049	(BW^0.25^/h)	Optimized to monkey
Tissue binding constants					
KAST	Association rate constant for striatum	0.182	0.182	(/µg/l*h)	Yoon *et al.* (2019)
KAOB	Association rate constant for olfactory bulb	0.182	0.182	(/µg/l*h)	Yoon *et al.* (2019)
KACB	Association rate constant for cerebellum	0.182	0.182	(/µg/l*h)	Yoon *et al.* (2019)
KAPT	Association rate constant for pituitary	0.182	0.182	(/µg/l*h)	Yoon *et al.* (2019)
KALIV	Association rate constant for liver	0.182	0.182	(/µg/l*h)	Yoon *et al.* (2019)
KALUNG	Association rare constant for lung	0.182	0.182	(/µg/l*h)	Yoon *et al.* (2019)
KABONE	Association rate constant for bone	0.182	0.182	(/µg/l*h)	Yoon *et al.* (2019)
KAOTH	Association rate constant for others	0.182	0.182	(/µg/l*h)	Yoon *et al.* (2019)
KDST	Dissociation rate constant for striatum	KAST*20 µg/l	KAST*20 µg/l	(/h)	Yoon *et al.* (2019), 20 or 25 µg/l dissociation constant
KDOB	Dissociation rate constant for olfactory bulb	KAOB*25 µg/l	KAOB*25 µg/l	(/h)	Yoon *et al.* (2019), 20 or 25 µg/l dissociation constant
KDCB	Dissociation rate constant for cerebellum	KACB*20 µg/l	KACB*20 µg/l	(/h)	Yoon *et al.* (2019), 20 or 25 µg/l dissociation constant
KDPT	Dissociation rate constant for pituitary	KAPT*20 µg/l	KAPT*20 µg/l	(/h)	Yoon *et al.* (2019), 20 or 25 µg/l dissociation constant
KDLIV	Dissociation rate constant for liver	KALIV*25 µg/l	KALIV*25 µg/l	(/h)	Yoon *et al.* (2019), 20 or 25 µg/l dissociation constant
KDLUNG	Dissociation rate constant for lung	KALUNG*25 µg/l	KALUNG*25 µg/l	(/h)	Yoon *et al.* (2019), 20 or 25 µg/l dissociation constant
KDBONE	Dissociation rate constant for bone	KABONE*25 µg/l	KABONE*25 µg/l	(/h)	Yoon *et al.* (2019), 20 or 25 µg/l dissociation constant
KDOTH	Dissociation rate constant for others	KAOTH*20 µg/l	KAOTH*20 µg/l	(/h)	Yoon *et al.* (2019), 20 or 25 µg/l dissociation constant
Maximal storage tissue capacities					
BMAXSTC	Striatum	102.38	102.38	(µg/l tissue)	Optimized to monkey
BMAXOBC	Olfactory bulb	329.89	329.89	(µg/l tissue)	Optimized to monkey
BMAXCBC	Cerebellum	437.04	437.04	(µg/l tissue)	Optimized to monkey
BMAXPTC	Pituitary	0.55	0.55	(µg/l tissue)	Optimized to monkey
BMAXLIVC	Liver	7906.44	7906.44	(µg/l tissue)	Optimized to monkey
BMAXLUNGC	Lung	165.46	165.46	(µg/l tissue)	Optimized to monkey
BMAXBONEC	Bone	189.76	189.76	(µg/l tissue)	Optimized to monkey
BMAXBODC	Rest of body (other)	227.86	227.86	(µg/l tissue)	Optimized to monkey
Fraction deposited and olfactory bulb translocation					
FDEPLU	Fraction deposited in lung	0.1396	0.2473	Unitless	MPPD ver. 3.04
FDEPNO	Fraction deposited in nasal olfactory region	0.4313*0.91	0.3762*0.95	Unitless	MPPD ver. 3.04; airflow split (Kepler *et al.* 1998; Schroeter *et al.* 2010)
FDEPNR	Fraction deposited in nasal respiratory region	0.4313*0.09	0.3762*0.05	Unitless	MPPD ver. 3.04; airflow split (Kepler *et al.* 1998; Schroeter *et al.* 2010)
KDEPLUC	Lung clearance to deep tissues	10.0	10.0	(BW^0.25^/h)	Schroeter *et al.* (2011)
KSHALLUC	Lung clearance to shallow tissues	100.0	100.0	(BW^0.25^/h)	Schroeter *et al.* (2011)
KNPOBC	Transport from nasal olfactory to olfactory bulb	0.0035	0.0035	(BW^0.25^/h)	Schroeter *et al.* (2011)
KDEPNRC	Uptake from the nasal respiratory	0.0012	0.0012	(BW^0.25^/h)	Schroeter *et al.* (2011)
GI parameters					
FDIETUP	Fraction diet intake bioavailable	2.00E-03	0.06	Unitless	Schroeter *et al.* (2011)
KGI	Rate constant of uptake from GI lumen to epithelium	0.06	0.0026	(/h)	Schroeter *et al.* (2011)
FENT	Fraction taken into GI epithelium	0.011	0.005	Unitless	Schroeter *et al.* (2011)
KENT	Rate constant of release from GI epithelium	0.0022	0.0022	(/h)	Schroeter *et al.* (2011)
KFECES	Fecal excretion rate constant	0.6	0.6	(/h)	Schroeter *et al.* (2011)
Biliary elimination flow and rates					
KBILEC	First order rate constant	0.051	0.051	(l/h/BW^0.75^)	Schroeter *et al.* (2011)
QBILE	Bile flow	0.00078	NA	(l/h/BW)	Schroeter *et al.* (2011)
Dose-dependent biliary induction constants					
KBINDUC	Maximal biliary induction factor	2.50	2.50	Unitless	Schroeter *et al.* (2011)
KM	Biliary affinity dissociation constant	0.027	0.027	(µg/l)	Schroeter *et al.* (2011)
SLOPE	Slope factor	3.00	3.00		Schroeter *et al.* (2011)

NA (not applicable): only used for monkey bile concentration; no bile concentration was predicted for human.

##### Monkey model parameterization

Physiological and anatomical parameters for the monkey PBPK model were obtained from Davies and Morris (1993), Dorman *et al.* (2006a), and Nong *et al.* (2009) (Table 1). The body weight of 2.5 kg corresponded to the average weight of the monkeys in Dorman *et al.* (2006a). For chemical-specific parameters, only the tissue influx (KIN), efflux (KOUT), and maximal binding capacities (BMAX) were re-estimated in this effort. The remaining chemical-specific parameters including those for the GI and biliary compartments, as well as association and dissociation constants were from Schroeter *et al.* (2011) or Yoon *et al.* (2019). Updated pulmonary region deposition fractions (see above) were treated as in the Schroeter *et al.* (2011) model where the MPPD (ver. 3.04) head deposition was allocated to the respiratory and olfactory region of the nose according to an airflow allocation of 9% olfactory, 91% respiratory (Kepler *et al.*, 1998). Significantly, a dose-dependent uptake process included in the globus pallidus and pituitary compartments in the earlier monkey models (Nong *et al.* 2009; Schroeter *et al.*, 2011) was not required to reproduce the monkey data with the revised model.

**Figure 3.**
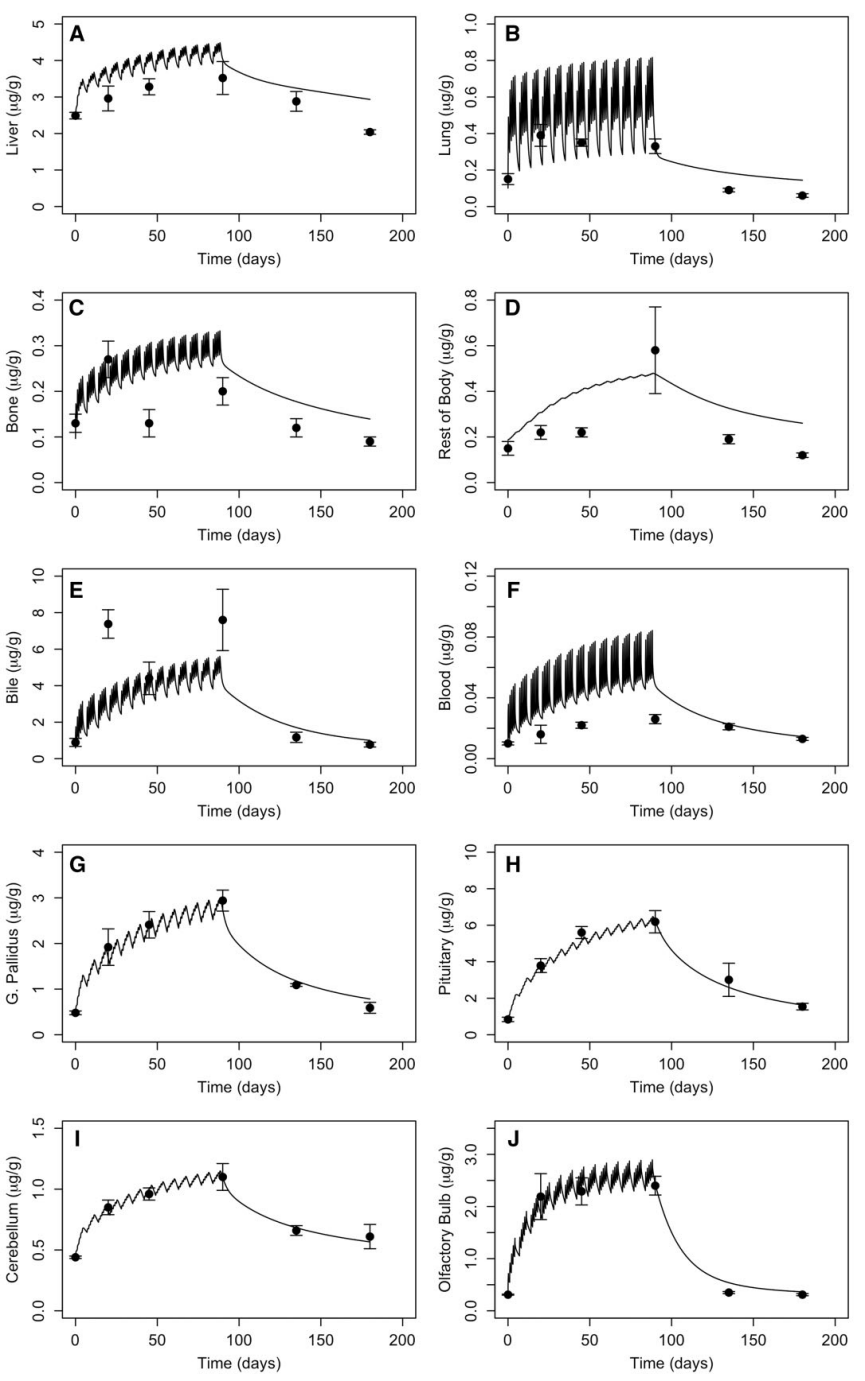
Simulated tissue Mn levels in monkeys exposed by inhalation to 1.5 mg Mn/m^3^ for 90 days (6 h/day, 5 days/week). The simulation results are compared with data from Dorman *et al.* (2006a). The curves are model simulations and symbols are means from 4 to 6 monkeys per exposure concentration.

**Figure 4.**
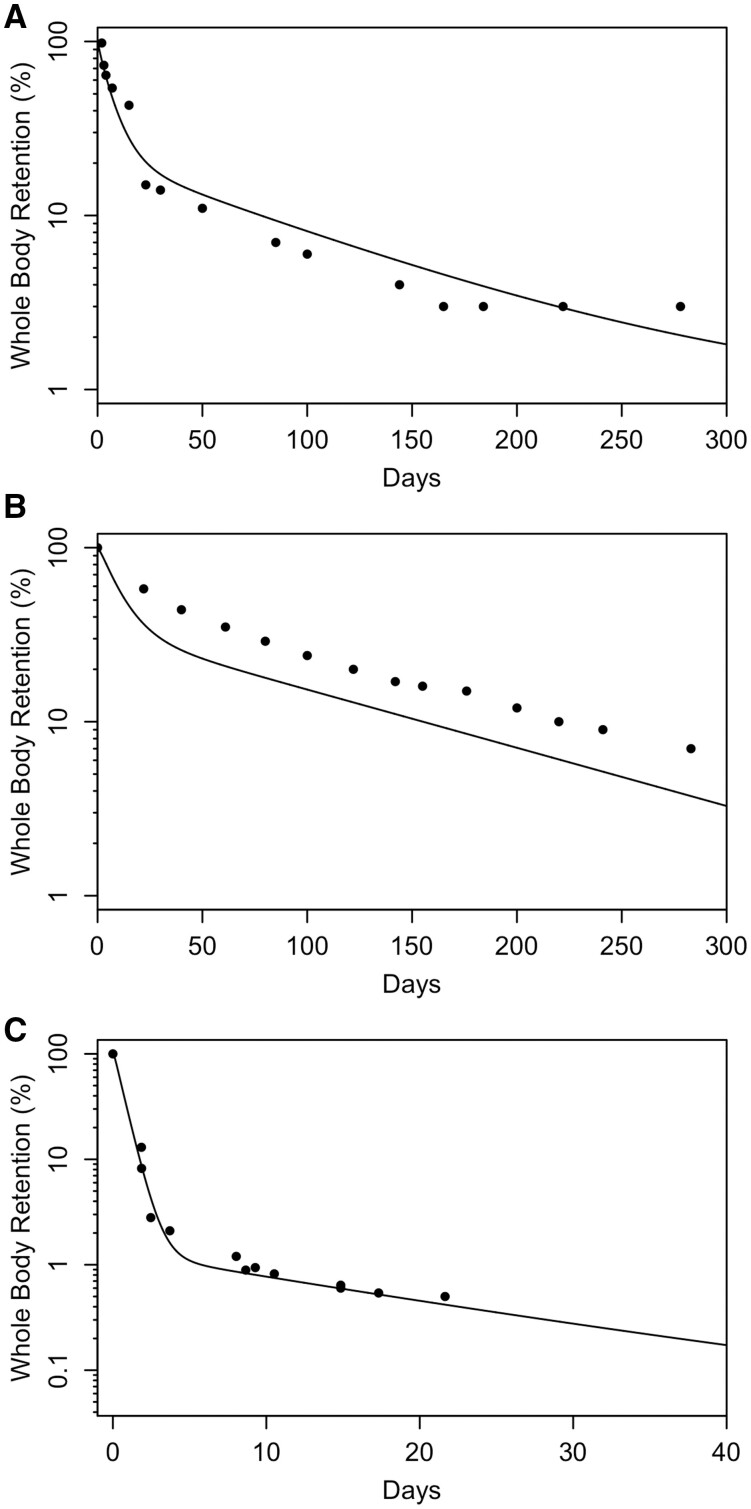
Simulated whole-body retention of ^54^Mn in monkeys compared with experimental data using of monkeys given a single dose of Mn via different exposure routes: (A) monkeys were exposed by ip injection to 200 µCi ^54^MnCl_2_ (Dastur *et al.*, 1971); (B) monkeys were exposed by iv administration to 0.6 µCi ^54^MnCl_2_ (Furchner *et al.*, 1966); (C) monkeys were orally dosed with 0.6 µCi of ^54^MnCl_2_ (Furchner *et al.*, 1966). Curves represent the model simulations, and the symbols are retention data from individual monkeys.

Parameter optimization for the monkey Mn transporter model was conducted to simultaneously estimate 3 parameters (KIN, KOUT, and BMAX) for each tissue. The cost function was based on minimization of the sum of squared error between the log model minus log data. The data sets used for optimization include the Dorman *et al.* (2006a) baseline diet values in all tissues (1st time-point in Figure 3A–J) and 1.5 mg/m^3^ inhalation time-course data (Figure 3A–J) and the whole-body clearance of ^54^Mn studies (Dastur *et al.* 1971—ip; Furchner *et al.* 1966—iv and oral; Figure 4A–C). Parameters were estimated using the nloptr package (Ypma *et al.*, 2020) which includes the derivation of the Subplex algorithm (NLOPT_LN_SBPLX) which is a variant of the Nelder-Mead algorithm. The final parameters are given in Table 2.

##### Human model parameterization

Physiological parameters in the human PBPK model were obtained from the literature (ICRP, 2002) (Table 1). As noted previously, Mn dietary intake can vary widely and is typically between 1 and 10 mg/day (ATSDR, 2000). An average dietary intake of 3 mg/day Mn, and dietary absorption (FDIETUP) and biliary excretion (KBILEC) were retained from Schroeter *et al.* (2011). As was done with the monkey, the binding and dissociation rates were set to provide rapid binding kinetics. The remaining chemical-specific parameters which had been optimized to the monkey kinetic studies (ie, tissue specific KIN, KOUT, and BMAX) were retained for simulation of the whole-body Mn clearance studies in humans.

##### Sensitivity analysis

A one-at-a-time (OAT) forward-difference sensitivity analysis was conducted to determine which model parameters had the greatest influence on the response variable. The sensitivity of all model parameters (excluding BW, QCC, and QPC) was assessed for the end of last exposure concentration in the globus pallidus. Both species were run with simulations assuming exposure for 90 days, with monkey and human exposed 6 h/day for 5 days/week. Normalized sensitivity coefficients (fractional change in output divided by fractional change in input) were calculated. Normalization for the response variable and the parameter was included to allow a comparison across parameters and doses. The output was deemed sensitive to a parameter if the resulting coefficient was >0.1 in absolute value. Only parameters that were influential on at least one output metric are reported.

##### Software

The acslX model from Schroeter *et al.* (2011) was translated into R using several open-source software packages including MCSim (version 6.0.2), R (version 4.0.3), RTools (version 4.0.0), and RStudio (1.1.463). The model was first translated into MCSim (Bois 2009) which allows for translation into C+. The C+ file is then compiled using the gcc compiler included in RTools to allow for integration with the vode algorithm included in the deSolve package (Soetaert *et al.* 2010), an R package. RStudio (ver. 1.0.136), which provides an integrated development environment for R, was used to edit model files and run simulations. The model code is included in the Supplementary Material.

## Results

###  

#### Monkey simulations

Simulation of the monkey studies used in the optimization of the influx and efflux rate constants and maximal binding capacities are shown in Figure 3A–J and Figure 4A–C. Overall, this recast Mn model provided reasonable fits to time-course tissue data (Figure 3A–J). This comparison highlights the relatively flat response of visceral organs liver (3A), lung (3B), bone (3C), and muscle (3D) along with the measured bile (3E) and blood (3F) concentrations reported by Dorman *et al.* (2006a). At the same time, the model captures the rapid rise during the 90-day repeated exposures, as well as the rapid clearance after the last exposure in the brain target tissues (3G and 3I) where very discrete portions of the brain were sampled, as well as the pituitary (3H) and olfactory bulb (3J).

In Dastur *et al.* (1971), 12 rhesus monkeys with an average body weight of 2.5 kg were administered an ip dose of 200 µCi of carrier-free ^54^MnCl_2_ solution. Whole-body activity of ^54^Mn was reported up to 278 days post-exposure. In keeping with the Schroeter *et al.* (2011) simulation of these data, the dietary intake was set to 80 ppm Mn, consistent with other published studies (Furchner *et al.*, 1966), and the baseline dietary uptake (0.0002) was used. The updated Mn model captured the nonlinear dose-dependence of whole-body clearance of ^54^Mn (Figure 4A). The same correspondence was also the case for the Furchner *et al.* (1966) whole-body clearance studies with ^54^MnCl_2_ which included single monkeys administered 0.6 µCi ^54^MnCl_2_ as either an iv (Figure 4B) or oral (Figure 4C) bolus where the updated model provides an excellent representation of the data.

**Figure 5.**
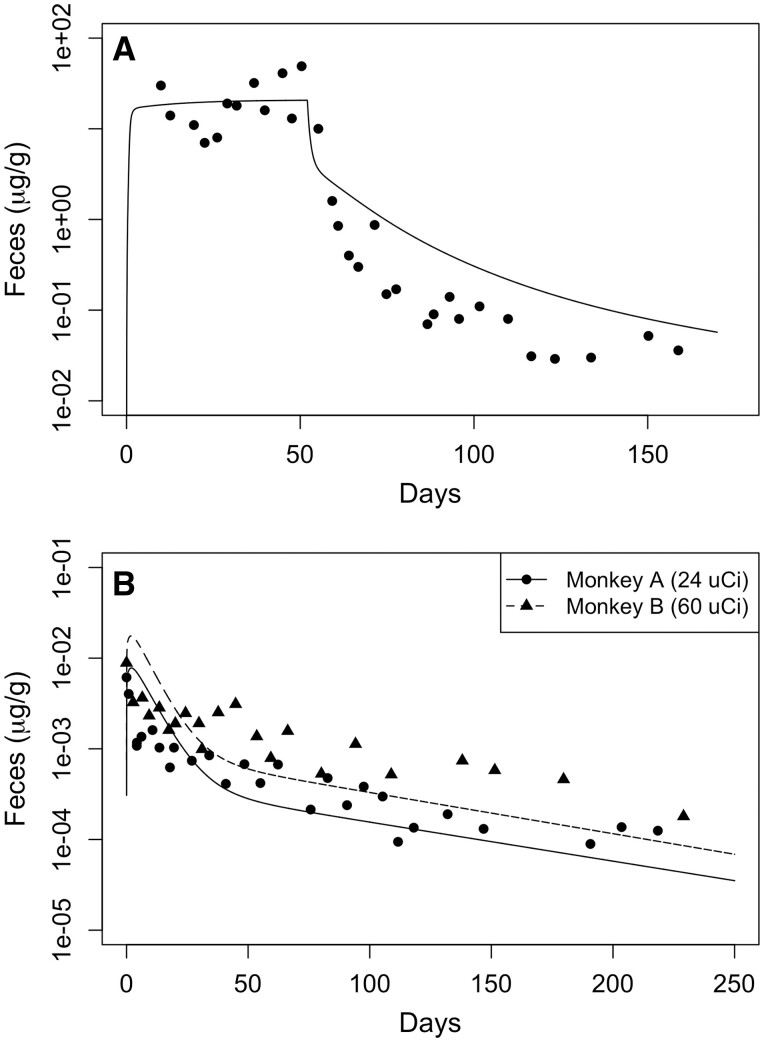
Simulated fecal excretion of ^54^Mn in monkeys compared with the experimental data from Newland *et al.* (1987): (A) one monkey was injected subcutaneously to 200 µCi ^54^Mn and 400 mg Mn in a MnCl_2_ solution; (B) two monkeys were exposed by inhalation to a nebulized aerosol of ^54^MnCl_2_. The curves represent model simulations, and the symbols are fecal excretion data from individual monkeys.

The optimized model was used to simulate the time-course fecal concentrations in monkeys after sc infusion (total infused: 200 µCi ^54^Mn and 400 mg Mn in a MnCl_2_ solution; Figure 5A) over 50 days and single exposure inhalation of nebulized ^54^Mn (monkey A: 24 µCi; monkey B: 60 µCi; Figure 5B) reported by Newland *et al.* (1987). Both these dose routes were well described by the PBPK model without any alteration of the Mn kinetic parameters. The model performs well in capturing the steady-state whole-body tracer Mn achieved during the infusion (Figure 5A) and, although there is some over-prediction of fecal concentrations during the clearance phase, the simulations provide a reasonable approximation of the shape of the clearance phase assuming approximately 50% of the dose was lost via necrosis of the skin reported at the pump injection sites. With these limitations, this result was considered a more qualitative simulation given the uncertainty in the administered dose in the sc study. Nonetheless, the simulation of the nebulized tracer Mn was accurately captured by the updated Mn model for monkey A, whereas monkey B, with the sc dosing, was only slightly underpredicted. Overall, the Mn model provides a good representation of the time course of fecal ^54^Mn elimination after single exposure inhalation or continuous sc infusion.

**Figure 6.**
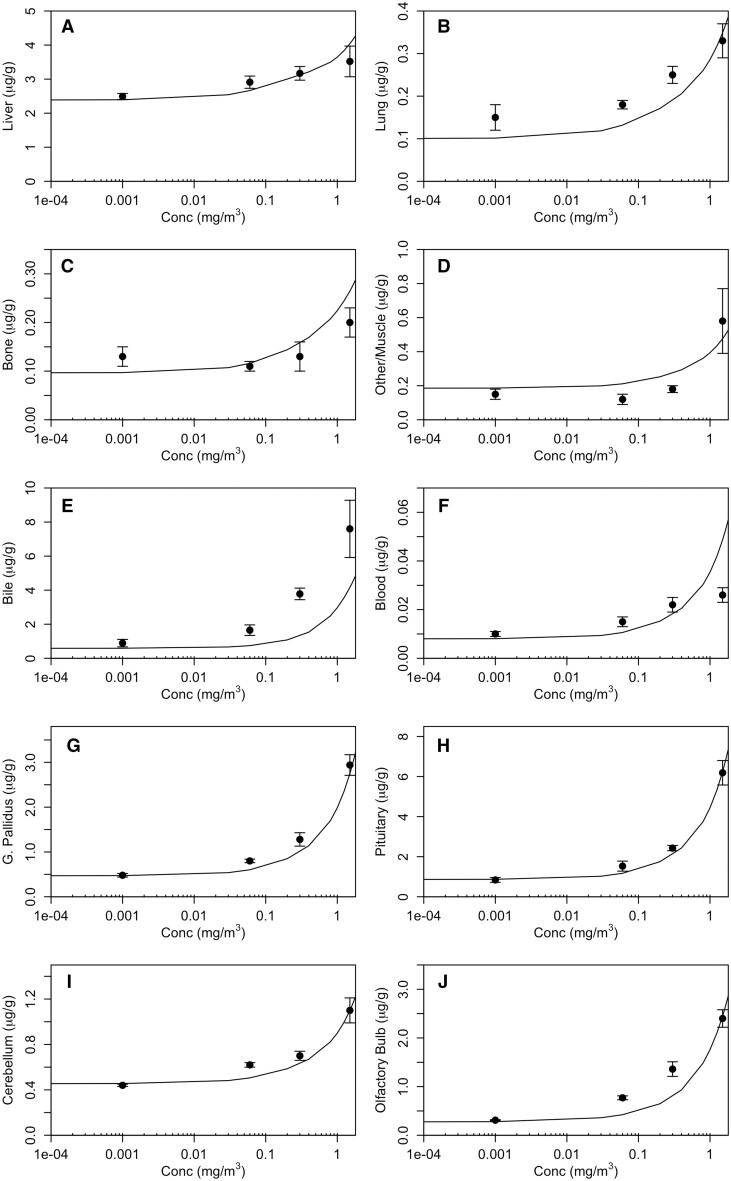
Simulated tissue Mn levels in monkeys exposed by inhalation to 0.0, 0.06, 0.3, or 1.5 mg Mn/m^3^ for 90 days (6 h/day, 5 days/week). The simulation results are compared with data from Dorman *et al.* (2006a). The curves are model simulated tissue concentrations at end of last exposure and symbols are means from 4 to 6 monkeys per exposure concentration.

Simulation of the tissue concentration response reported by Dorman *et al.* (2006a) after 90 days of inhalation exposure (6 h/day, 5 days/week) is shown in Figures 6A–J. The updated model provides very good fits to the concentration response across all tissues, capturing both the relatively flat response region and the more pronounced increases above about 0.1 mg/m^3^. In all tissues, the Mn model captured the changes in Mn disposition and clearance due to the increasing exposure concentration where saturation of tissue binding sites will be followed by marked increases in free Mn concentrations.

#### Human simulations

**Figure 7.**
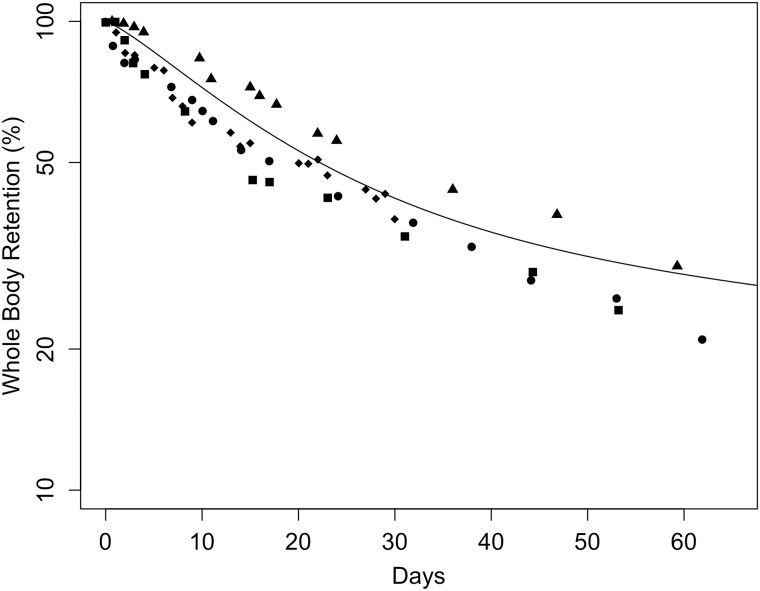
Comparison of simulated whole-body retention in humans given an iv dose of ^54^Mn with the whole-body retention data of Mahoney and Small (1968) and Mena *et al.* (1967). The curve represents the model simulation; the symbols for the Mahoney and Small (1968) study are retention data for individual subjects and the symbols for the Mena *et al.* (1967) study represent average retention from 8 subjects (square: subject C.H.; circle: subject M.M.; triangle: subject H.H.; diamond: Mena average).

**Figure 8.**
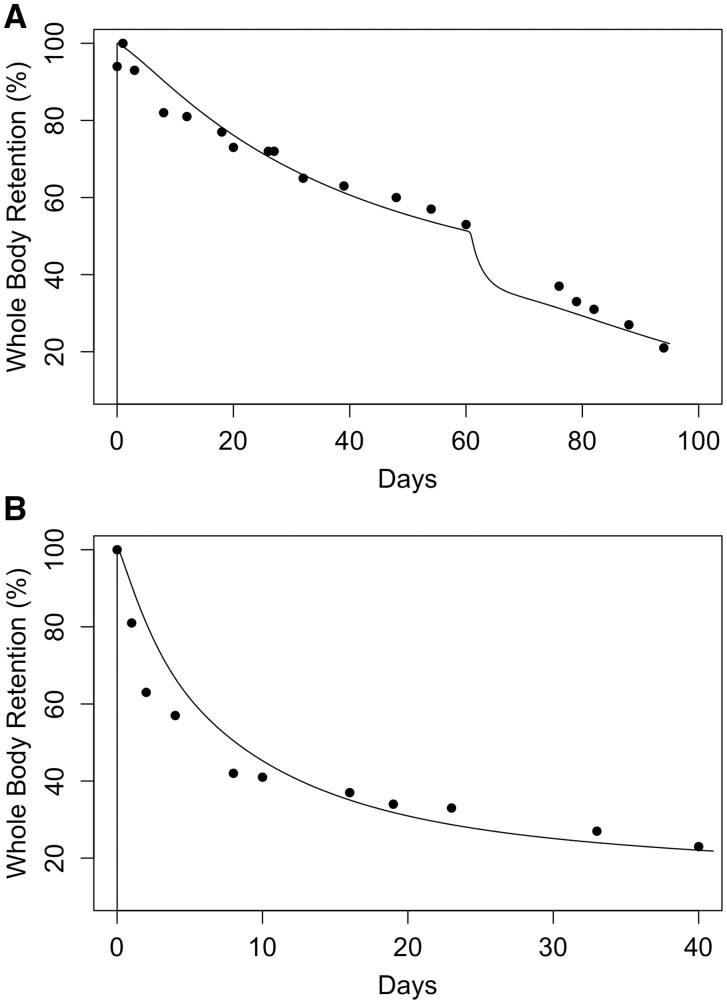
Comparison of simulated whole-body retention of ^54^Mn in human volunteers given supplemental Mn compared with the experimental data from Mahoney and Small (1968): (A) subject J.M. was on a reduced calorie diet and began to ingest 800 mg/day Mn on day 60 of the study; (B) subject W.S. was preloaded with 300 mg/day Mn 10 days prior to start of the study. The curves represent model simulations, and the symbols are retention data from individual subjects.

**Figure 9.**
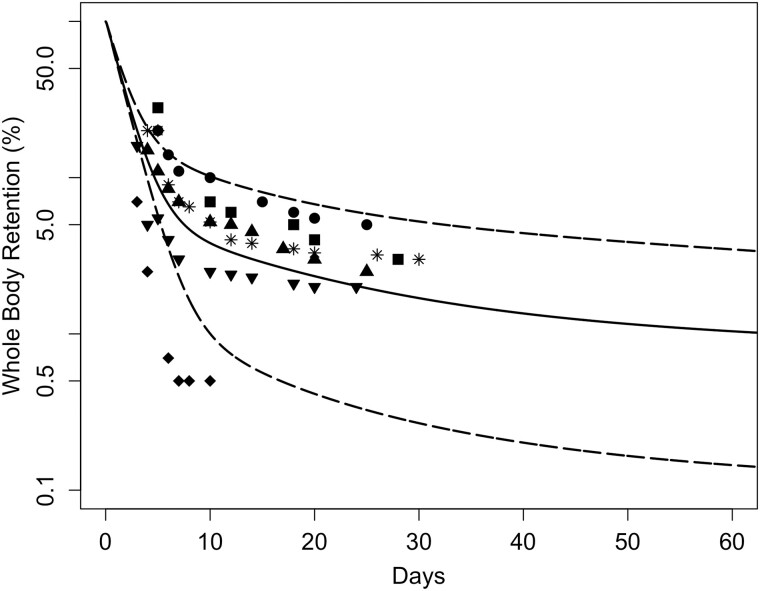
Comparison of simulated whole-body retention of ^54^Mn in humans with the experimental data from Davidsson *et al.* (1988), where human volunteers ingested a meal labeled with ^54^Mn. The curve represents a model simulation, and the data symbols are from individual subjects. The dashed curves represent the effect of simultaneous 3-fold opposed changes in FDIETUP and KBILEC, to indicate the sensitivity of these 2 parameters for predictions of the whole-body clearance of ^54^Mn (solid line: FDIETUP = 0.06 and KBILEC = 0.051 l/h/BW^0.75^, upper dashed line: FDIETUP = 0.18 and KBILEC = 0.017 l/h/BW^0.75^, lower dashed line: FDIETUP = 0.02 and KBILEC = 0.153 l/h/BW^0.75^).

**Figure 10.**
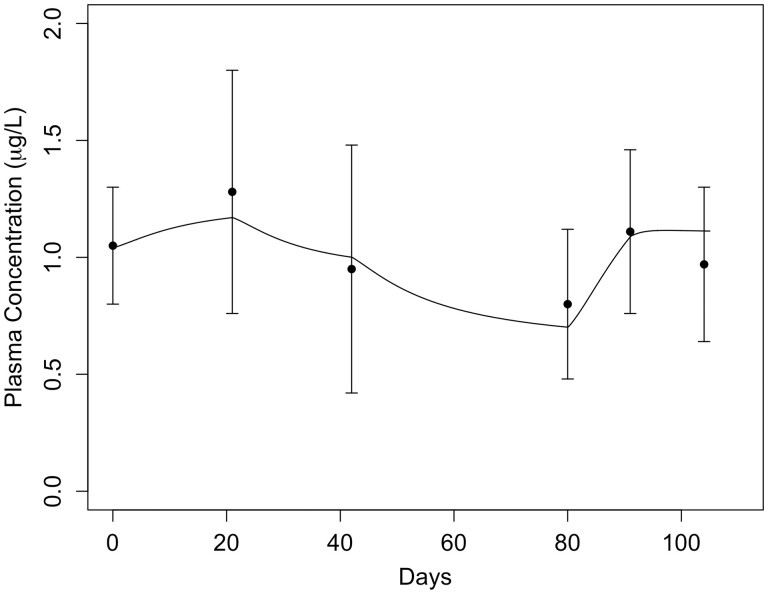
Comparison of simulated plasma Mn concentrations with the experimental data from Freeland-Graves (1994). Simulated plasma Mn concentrations were calculated as 20% of simulated blood Mn concentrations. The data points represent the average plasma Mn concentration from 5 healthy men consuming diets containing different levels of dietary Mn (mean ± SD): Day 1–21: 2.36, Day 22–42: 2.89, Day 42–63: 2.06, Day 64–101: 1.21, Day 102–112: 3.79, Day 113–125: 2.65 mg Mn/day.

For the human modeling, the available data are primarily ^54^Mn studies of whole-body retention (Figures 7–9) or changes in plasma concentration associated with changes in dietary intake (Figure 10). As discussed previously, the parameterization for the human was primarily based on the monkey optimized parameters for maximal tissue binding, the rapid association/dissociation of tissue Mn binding, and more rapid transport into and out of tissues described for the adult rat Mn model (Yoon *et al.*, 2019). Only the fraction uptake of dietary Mn and biliary excretion rate were estimated by fitting the model to the human data. All the model parameters reported by Schroeter *et al.* (2011) were retained in the human model.

Figure 7 shows the comparison of simulated whole-body retention in humans given an iv dose of ^54^Mn with the whole-body retention data of Mahoney and Small (1968) and Mena *et al.* (1967). The updated model provides an excellent fit to the initial whole-body retention of Mn up to 40 days post administration of the tracer Mn. Although the Mn model appeared to transition to a slower clearance phase somewhat earlier than seen in the data, the simulation was within a factor of 2 for both the Mahoney and Small (1968) and Mena *et al.* (1967) measured whole-body retention.

Simulation of the whole-body retention in subjects administered supplemental Mn (Mahoney and Small, 1968) is shown in Figure 8. The subject (J.M.) in Figure 8A was on a low-calorie diet and received about 1/3 of the daily intake of Mn prior to the iv administration of ^54^Mn and for the first 60 days after tracer dosing. On day 60, the subject received daily supplementation of Mn (800 mg/day) for the remainder of the study. As with the Schroeter *et al.* (2011) model, to simulate the study results through 60 days, FDIETUP was increased from 0.06 to 0.062 and KBILEC was decreased from 0.051 to 0.02 l/h/BW^0.75^. On day 60 of the simulation, FDIETUP was returned to 0.06. Figure 8B shows the simulation of subject W.S. who was administered a daily supplemental oral dose of Mn (300 mg/day) starting 10 days prior to the iv administration of ^54^Mn and was continued throughout the study. As described in Schroeter *et al.* (2011), FDIETUP was reduced each day for the first 15 days (ie, −day 10 to +day 5). For our simulation, the best fit to the whole-body retention on Mn in subject W.S. was achieved with a daily reduction of 22% when compared with the 26% per day reduction in FDIETUP reported by Schroeter *et al.* (2011).

The whole-body retention of ^54^Mn administered orally in a single meal (Davidsson *et al.* 1988) to 6 adult females is shown in Figure 9. As seen with the iv-tracer study, the simulations were generally consistent with data in 5 of the 6 subjects. One subject showed faster elimination of tracer and may have been on a diet with higher Mn levels than the other participants. The dashed curves in Figure 9 demonstrate the impact of a 3-fold opposed change in FDIETUP and KBILEC, to illustrate the influence of these 2 parameters on the whole-body clearance of ^54^Mn. (solid line: FDIETUP = 0.06 and KBILEC= 0.051 l/h/BW^0.75^, upper dashed line: FDIETUP = 0.18 and KBILEC = 0.017 l/h/BW^0.75^, lower dashed line: FDIETUP = 0.02 and KBILEC = 0.153 l/h/BW^0.75^).

Overall, the current PBPK model simulations were consistent with results for all the data from human volunteers, capturing the clearance of orally administered tracer Mn over the 30-day simulation (Figure 9) and with the iv-tracer study (Figure 7). The Mn model captured the transition from the rapid clearance phase to a slower phase seen at approximately 10 days—a result consistent with the Davidsson *et al.* (1988) retention data. Simulated plasma Mn concentrations (Figure 10) were also compared with plasma Mn measurements from men consuming different levels of dietary Mn (Freeland-Graves, 1994). The updated Mn model successfully captured the change in plasma concentration associated with the change in dietary Mn intake and is an improvement over the simulations from Schroeter *et al.* (2011), who used a model with much slower association/dissociation rate constants for tissue binding of Mn. The simulations in Figure 10 did not require changing FDIETUP with changes in dietary Mn due to the small change in diet over the study.

#### Sensitivity analysis

OAT sensitivity coefficients for predicted globus pallidus concentration from a 1% change in model parameter values were determined at the end of 90 days (6 h/day, 5 days/week) for inhalation exposure concentrations of 0.01, 0.1, and 1.0 mg/m^3^ Mn in monkeys and humans (Table 3). Model predictions were most sensitive to the influx and efflux diffusion rate constants (all parameters starting with “KIN” and “KOUT”). Model predictions were also sensitive to dietary absorption (FDIETUP and INFAC) and biliary elimination (KBILEC), although predictions became less sensitive with increasing inhalation exposure concentration as brain Mn levels were driven more by inhalation than diet. Sensitivity of model predictions displayed similar trends in monkeys and humans and were similar with sensitivity results in monkey and human reported in Schroeter *et al.* (2011) apart from the additional tissue transporter parameters (KINLIVC and KOUTLIVC) for liver that were added as part of Mn model refinement.

**Table 3.** Sensitivity analysis (one at a time^a^) of peak Mn concentrations in the globus pallidus of monkeys and humans at inhalation concentrations of 0.01, 0.1, and 1.0 mg/m^3^

	Normalized sensitivity coefficient^a^					
	Monkey			Human		
Exposure (mg/m^3^)	0.01	0.1	1	0.01	0.1	1
Model parameter						
KBILEC	−0.66	−0.50	−0.31	−0.67	−0.56	−0.29
VLIVC	NS	−0.11	−0.45	NS	−0.11	−0.39
VSTMC	−0.80	−0.85	−0.94	−0.71	−0.78	−0.92
FDEPLU	NS	NS	0.25	NS	0.19	0.41
FDEPNR	NS	0.17	0.45	NS	0.14	0.28
KDEPNRC	NS	NS	0.18	NS	NS	0.18
KINSTC	0.81	0.86	0.95	0.71	0.79	0.93
KINLIVC	−0.67	−0.61	−0.75	−0.67	−0.67	−0.68
KOUTSTC	−0.80	−0.84	−0.90	−0.70	−0.76	−0.88
KOUTLIVC	0.66	0.50	0.31	0.68	0.57	0.29
BMAXSTC	0.20	0.15	NS	0.31	0.22	NS
′KBINDUC	NS	NS	−0.20	NS	NS	−0.16
KM	0.13	0.23	0.15	NS	NS	0.20
SLOPE	0.15	0.19	NS	NS	NS	NS
FDIETUP	0.64	0.36	NS	0.73	0.48	NS
INFAC	0.63	0.36	NS	NS	NS	NS

NS, not sensitive. Sensitivity coefficients (normalized to parameter and endpoint) reflect a 1% change in model parameters.

a One at a time sensitivity coefficient was assessed using the forward difference with a delta of 1%. Coefficients were normalized to the parameter and endpoint to allow comparison across parameters and inhalation concentrations. Only parameters with at least 1 coefficient >0.1 were considered sensitive. Both monkey and human were exposed for 90 days, 6 h/day, and 5 days/week. The globus pallidus concentration at the end of the last exposure was used in the calculation.

## Discussion

###  

#### Kinetics of Mn interactions in tissues

The Schroeter *et al.* (2011) monkey and human Mn PBPK model structure was based on a particular set of assumptions and coherently integrated the state of the Mn biology at the time. As with these original models, the updated Mn model structure for the rat (Yoon *et al.*, 2019) provided time profiles in the striatum after inhalation that were predominantly determined by increase in free Mn, with free tissue Mn expected to be responsible for Mn toxicity. However, the processes that account for control of free Mn in tissues differ between these models.

Yoon *et al.* (2019) updated Mn disposition in tissues with a description that is more consistent with current understanding of Mn biology. The dissociation equilibrium constant, *K*_D_, was a similar value across tissues, consistent with common biological functions of Mn irrespective of tissue/cell type. The *K*_D_ used in the updated model (approximately 0.5 μM) reflected general binding affinity of Mn for multiple binding sites within tissues. To date there is little direct information to inform the amounts or affinity of the binding sites. Limited experimental evidence reports cellular free Mn in rat hepatocytes at around 0.2–1 μM based on electron paramagnetic resonance analysis (Ash and Schramm, 1982; Powell and Brew, 1976). Additionally, experimental studies report dissociation constants for Mn binding to galactosyltransferase and Mn^2+^-ATPase as 2.0 and 0.88 μM, respectively (Grisham and Mildvan, 1974; Powell and Brew, 1976). Our fitted dissociation rate constants, set at either 0.37 or 0.46 µM (20 or 25 µg/l) depending on the tissue, are in line with the estimated cellular concentration of free Mn and consistent with the *K*_D_ from Yoon *et al.* (2019) and this literature. The dissociation rate constants, equivalent to half-lives of 4–5 min, are consistent with more readily interchangeable forms of cellular and subcellular forms of Mn. As in the earlier models, the binding capacity was set based on tissue concentrations measured in control animals or in human cadavers. The ability of the association/dissociation constants from the adult rat Mn model to represent the available data in the monkey is evidence that the primary binding depots are similar across mammalian species. Although the present model has much faster dissociation rate constants than used previously, these rate constants may not directly relate to dissociation of Mn from specific binding partners, such as Mn-requiring proteins throughout the cell. Rather they are likely to include both cytoplasmic protein partner dissociation rates and transport processes related to efflux of cellular Mn from subcellular organelles which also contain various metal ion transporters (Kambe *et al.*, 2021). There remains uncertainty in the specific values of *k*_A_ and *K*_D_. We found no literature reporting these kinetic constants and, due to the large number of binding partners, it would be difficult to generalize from results with any one protein to all other Mn-binding proteins. Upper bounds could be estimated by varying *k*_D_ and *k*_A_ to see what values would still fit the various data sets. No such exercise was attempted in this article.

#### Scaling to humans

The approach taken to extend the monkey model to the human in this effort differs from the approach used in Schroeter *et al.* (2011). In Schroeter *et al.* (2011) human model, some tissue-specific Mn parameters (KIN, KOUT, and BMAX) from the monkey were adjusted to provide better agreement with human cadaver tissue data and whole-body clearance studies resulting in increased uncertainty in the human model. For this updated monkey/human Mn model, only the uptake and clearance parameters, which are informed by the whole-body retention studies, were retained from the Schroeter *et al.* (2011) human model. The parameters associated with tissue binding and diffusion were scaled allometrically from the revised monkey model, which reflect the more rapid association/dissociation processes. With the success of this extrapolation in fitting the various human studies, the uncertainty in the Schroeter *et al.* (2011) human Mn model is significantly reduced because the parameters are now based on data that are informative of the ready availability of Mn among tissue stores. This result also supports the conclusion that the tissue disposition of Mn is conserved across species, with characteristics of the control of uptake of dietary Mn and biliary excretion accounting for many of the observed differences in whole-body uptake and clearance. The importance of control of dietary uptake and elimination are evident in fitting human tracer studies (Figure 9) where individual curves could be fitted by relatively small adjustments in uptake parameters for dietary Mn. Although there is no information on changes in human tissue levels with increasing exposures, the consistency of basal tissue levels across species and correspondence of altered whole-body clearance and increasing tissue levels in monkeys, provides high confidence that results from monkeys on tissue levels provide a good indication of conditions that will lead to similar increases in humans.

#### Parameter estimation

Compared with PBPK model development for the rat (Yoon *et al.*, 2019), the equivalent monkey model was developed with more state-of-the-art curve fitting for the various data sets. With use of this more rigorous fitting approach, it was still necessary to include induction of the rate constant for biliary excretion with increases in exposure concentration and the induction parameters were used as described in Schroeter *et al.* (2011). Although biliary clearance was induced with increasing blood levels of Mn in the current monkey model, there was no need to retain the dose-dependent increase in brain region uptake as had been done in Schroeter *et al.* (2011). This difference in uptake processes, ie, inducible versus noninducible brain uptake, in the 2 monkey models may be due to the use of more formal fitting methods in the current modeling, together with inclusion of rapid association/dissociation processes that allow more rapid adjustments of tissue concentrations at initiation or cessation of exposures.

#### Uptake and clearance processes for cellular manganese

Although recasting the Mn PBPK models with rapid association and dissociation processes permits more rapid adjustment of tissue manganese with abrupt changes in oral or inhalation intakes (Yoon *et al.*, 2019), these models still rely on fitting tissue uptake and elimination using input and efflux clearances (Table 2). Divalent metals are moved from blood into tissues and exit tissues to blood via transporters. They also move from the cytosol into subcellular organelles, each with differing requirements for the metal. Perhaps, the most well-characterized metal in relation to transporter proteins in cellular compartments is zinc (Kambe *et al.*, 2021), where over 20 transporters have been described controlling uptake and efflux from blood into tissues and uptake and efflux from cytosol into subcellar organelles. These transporters fall into 2 broad categories, ZnT proteins and ZIP family proteins, with the former serving as efflux transporters and the latter as uptake transporters. The ZnT efflux proteins are members of the Slc30A1 through A10 family and the ZIP uptake proteins are in the Slc39A1 through Slc39A14 family. Inherited mutations in Slc30A10 (Quadri *et al.*, 2012) and Slc39A14 (Tuschl *et al.*, 2016) cause familial Mn neurotoxicity, and mutations in Slc39A8 cause low blood Mn concentrations, impaired glycosylation, and severe developmental abnormalities (Park *et al.*, 2015). Clearly, these various transporter proteins have important functional roles in Mn homeostasis. These inherited mutations demonstrate both the high dose toxicity and essentiality based on the observation with loss of function of Slc39A8.

More detailed studies of the importance of Mn transporters in Mn toxicity have been accomplished using whole-body and tissue-specific knock-out mice with Slc30A10 and Slc39A14 (Mercadante *et al.*, 2019). Whole-body knock-out of Slc39A10 led to accumulation of manganese in brain while pan-glial knockouts or the liver-specific knockout showed no significant tissue accumulation. Knocking out Slc30A10 in liver and enterocytes caused increased tissue Mn, but the increases were not as large as those seen with the global knock-out (Mercadante *et al.*, 2019). Similarly, knock-out of Slc39A14 in liver did not lead to increases in tissue Mn as large as those seen with the whole-body knock out. At first glance, it seems paradoxical that increased tissue Mn occurs with loss of an efflux transporter (Slc30A10) and an uptake transporter (Slc39A14). However, the reason that loss of either causes Mn toxicity is likely associated with the roles of each transporter in biliary elimination of Mn. Net transport from blood to bile requires Slc39A14 transport from blood into hepatocytes and then efflux from hepatocytes to bile via Slc30A10 (Prajapati *et al.*, 2021). The role of these and other transporters in uptake of Mn from the diet into enterocytes and efflux from enterocytes to blood is not as well-defined.

This rapid association-dissociation model for cellular Mn described here simply uses aggregated clearance terms for uptake into and efflux out of tissues without identifying specific molecular determinants of the movement. Nonetheless, for the purposes of Mn risk assessment, this model recapitulates conditions leading to brain accumulation of Mn in monkeys and characteristics of whole-body kinetics in humans and provides a basis for estimating the inhalation exposures that will cause significant increases in Mn in target areas of the brain. Because of the improved representation of Mn binding and movement compared with older modeling efforts (Nong *et al.*, 2009; Schroeter *et al.*, 2011), these newer model structures are more biologically realistic, but both the older models and the current model give similar results for the dose response of tissue accumulation and Mn intake. One clear difference in fitting time course Mn studies was the ability of the model, with more rapidly exchangeable tissue stores, to better represent whole body elimination of tracer ^54^Mn after cessation of inhalation exposures at various concentrations (Yoon *et al.*, 2019; Figs. 2 and 3).

## Conclusion

PBPK models with more rapid association and dissociation kinetics of bound Mn better represent the known biochemistry of Mn compared with earlier models with much more slowly exchangeable Mn. This revised model structure was applied to describe dose dependencies of Mn in tissues of monkeys following inhalation exposures. The monkey model was also scaled to humans and was consistent with various human data sets evaluating clearance of tracer doses of ^54^Mn. Despite the much improved fidelity with Mn biology of this revised model structure, both the older and newer PBPK models adequately described the dose dependence of increasing Mn concentration in various brain regions with increasing inhalation exposures or with increasing dietary intakes. Further mechanistic studies of Mn transporters, especially in regulating the dose dependencies in intestinal uptake and biliary excretion, should improve the correspondence between transporter properties and the fitted clearance terms that regulate tissue uptake, tissue efflux, and biliary excretion in the present models. Even absent further mechanistic development, the current PBPK models are well-suited for assisting in risk assessments of manganese.

## Supplementary Material

kfac123_Supplementary_Data
