# Supplementary material for: Incorporation of rapid association/dissociation processes in tissues into the monkey and human physiologically based pharmacokinetic models for manganese
Source: Toxicol Sci. 2022 Dec 1;191(2):212–26. doi: 10.1093/toxsci/kfac123 (PMC9936208; doi:10.1093/toxsci/kfac123)
Supplement: kfac123_Supplementary_Data [file kfac123_supplementary_data.docx]

Supplemental Information

Table S1. Mean whole brain and regional brain weights, SD, fraction of brain and BW from all animals in the Dorman et al. (2006a) study (n=36).

|  | Mean (g) | SD | Fraction of Brain* |
| --- | --- | --- | --- |
| Brain | 97.95 | 7.32 | 0.037 |
| Pituitary | 0.036 | 0.01 | 0.00037 |
| Right olfactory bulb | 0.027 | 0.007 | 0.00056 |
| Right olfactory tract | 0.014 | 0.006 | 0.00028 |
| Right olfactory cortex | 0.26 | 0.086 | 0.0053 |
| Right caudate | 0.52 | 0.11 | 0.011 |
| Right putamen | 0.51 | 0.15 | 0.010 |
| Right globus pallidus | 0.11 | 0.050 | 0.0022 |
| Right trigeminal nerve | 0.054 | 0.013 | 0.0011 |
| Right cerebellum | 4.18 | 0.52 | 0.085 |
| Right frontal cortex | 1.55 | 0.70 | 0.032 |
| Right rest of brain | 41.79 | 3.42 | 0.85 |
| BW (kg) | 2.61 | 0.31 |  |

*For tissues with only right hemisphere reported, the fraction of brain was doubled assuming that the left hemisphere would have equal weight.

Code for the monkey/human MN PBPK model

Note: parameters are set in simulation scripts

# Manganese PBPK model for monkey and human

# The model inputs concentrations in ug/ml, diet in ppm and inhalation

# in mg/m3. It converts all inputs to ug or ug/hr for the calculations.

# After integrating to account for time dependent changes, the tissue concentrations

# are calculated as ug/ml for comparisons with data sets. Tracer kinetics simulate

# whole body Mn54 (kBq) based on exposure in Ci, assuming that tracer mass is negligeable.

#

#

# - Conversion of Schroeter monkey/human PBPK model for Mn from acslX to R - Jerry Campbell - 2/3/2022

# - Updated tissue:blood exchange for Mn to transporter description (Yoon et al. 2019) in lieu of partitioning

# - removed concentration related changes in brain tissue influx (G. Pallidus and Cerebellum)

States = {ADEPLU,ALUBD,ALUNG,BMNLUNG,ADEPNR,ANOSE,ANOLF,ABILE,ALBD,AL,BMNLIV,AGL,AGE,FECES,ALGL,ABRBD,ABRST,BMNST,

ABROB,BMNOB,ABRCB,BMNCB,ABRPT,BMNPT,ABOBD,ABONE,BMNBONE,AOBD,AO,BMNOTH,AART,AVEN,ASQ,

XADEPLU,XALUBD,XALUNG,XBMNLUNG,XANOSE,XAIP,XALBD,XAL,XBMNLIV,XAGL,XAGE,XALGL,XFECES,XABRBD,XABRST,XBMNST,

XABROB,XBMNOB,XABRCB,XBMNCB,XABRPT,XBMNPT,XABOBD,XABONE,XBMNBONE,XAOBD,XAO,XBMNOTH,XAART,XAVEN,XASQ,

LOSS,DOSE,DOSE54,XLOSS};

Outputs = {xdin,dietary,exposure,inhalation,kivr,intravenous,kdietr,atotlung,ctotlung,atotnose,ctotnose,atotliv,

ctotliv,cbile, ast,ctotst,aob,ctotob,acb,ctotcb,apt,ctotpt,abr,cprotmnbr,atotbr,ctotbr,atotbone,ctotbone,

atototh,ctototh,atotgut,atotbody,mass,xatotlung,xctotlung,xatotliv,xctotliv,xast,xaob,xacb,xapt,xabr,

xbmnbr,xatotbr,xatotbone,xctotbone,xatototh,xctototh,xatotgut,atot54,atotmn54,xrfecesmgtotal,qbalance, vbalance,

balance,balance54,ret54,CBRST,CBMNST,BST,CBRPT,CBRCB,CBROB,CART,CBILE,KDIET, KINHLU, KINHNR, KINHNO, XCART,

RADI, CLIV, KBILEX, KBILEINC, CVL, PCBLUNG, PCBLIV, PCBST, PCBOB, PCBCB, PCBPT, PCBBONE, PCBOTH, BOB,

VSTM, VCBM, VOFB, VPIT, VBLD, VBONE, VBRN, VLIV, VLUNG, VOTH,VNOLF, VNRESP ,VNOSE,

QBONE, QBRN, QLIV, QOTH, QNOSE};

Inputs = {XDIN,EXPOSURE,KIVR,KIPR,KINHR,KDIETR,KSUBQ,KSUBQR};

#************************************

# Physiological parameters

#************************************

#Blood Flows (fraction of cardiac output from Brown)

QCC = 99.9; # Cardiac output (L/hr/kg)

QPC = 99.9; # Alveolar ventilation (L/hr/kg)

QBONEC = 99.9; # Bone

QBRNC = 99.9; # Brain

QLIVC = 99.9; # Liver

QNOSEC = 99.9; # Nose (Frederick TAP 1998)

#Biliary elimination flow and rates

KBILEC = 99.9; # First order rate constant (L/hr/kg^0.75)

QBILEC = 99.9; # Bile flow (L/hr/kg)

#Fractional issue Volumes (fraction of body weight from Brown)

BW = 99.9; # Body weight (kg)

VBLDC = 99.9; # Blood

VBONEC = 99.9; # Bone

VBRNC = 99.9; # Brain

VLIVC = 99.9; # Liver

VLUNGC = 99.9; # Lung

# Fractional brain region volume (Dorman ToxSci 2001)

VSTMC = 99.9; # striatum

VCBMC = 99.9; # cerebellum

VOFBC = 99.9; # olfactory bulb

VPITC = 99.9; # pituitary (monkey)

# Nasal surface areas (Schroeter ToxSci 2008)

SANOLF = 99.9; # surface area nasal olfactory (cm2/kg)

SANRESP = 99.9; # surface area nasal respiratory (cm2/kg)

THNOSE = 99.9; # thickness nasal tissue (um)

#************************************

# Chemical specific parameters

#************************************

# Fractional depositions and clearances (estimated from MPPD and Brenneman ToxSci 2006)

FDEPLU = 99.9; # regional deposition in lung (pulmonary + tracheabronchiol)

FDEPNO = 99.9; # regional deposition in nasal cavity (olfactory)

FDEPNR = 99.9; # regional deposition in nasal cavity (respiratory)

KDEPLUC = 99.9; # transport from epithelium to lung tissue (/hr)

KSHALLUC = 99.9; # systemic uptake from lung epithelium (/hr)

KDEPNRC = 99.9; # systemic uptake from nasal respiratory (/hr)

KNPOBC = 99.9; # transport from olfactory to brain (/hr)

# permeablity diffusion flux constants (fitted with data)

#(kg^-0.25/hr)

KINSTC = 99.9; # striatum influx rate constant

KINCBC = 99.9; # cerebellum influx rate constant

KINOBC = 99.9; # olfactory bulb influx rate constant

KINPTC = 99.9; # pituitary influx rate constant

KINLUNGC = 99.9; # Lung influx rate constant

KINLIVC = 99.9; # Liver influx rate constant

KINBONEC = 99.9; # Bone influx rate constant

KINOTHC = 99.9; # Rest of body (Other) influx rate constant

#(kg^-0.25/hr/kg Tissue)

KOUTSTC = 99.9; # striatum efflux rate constant

KOUTCBC = 99.9; # cerebellum efflux rate constant

KOUTOBC = 99.9; # olfactory efflux rate constant

KOUTPTC = 99.9; # pituitary efflux rate constant

KOUTLUNGC = 99.9; # Lung efflux rate constant

KOUTLIVC = 99.9; # Liver efflux rate constant

KOUTBONEC = 99.9; # Bone efflux rate constant

KOUTOTHC = 99.9; # Rest of body (Other) efflux rate constant

#Tissue binding constants (fitted with data)

# ka unit changed, 2/4/2013, equations accordingly changed to reflect this correction

KDLIV = 99.9; #Dissociation constant for Liver (/hr)

KDOTH = 99.9; #Dissociation constant for Others (/hr)

KDBONE = 99.9; #Dissociation constant for bone (/hr)

KDLUNG = 99.9; #Dissociation constant for lung (/hr)

KALIV = 99.9; #Association constant for Liver (/ug/L/hr)

KAOTH = 99.9; #Association constant for Others (/ug/L/hr)

KABONE = 99.9; #Association constant for bone (/ug/L/hr)

KALUNG = 99.9; #Association constant for lung (/ug/L/hr)

KDST = 99.9; #Dissociation constant for striatum (/hr)

KDOB = 99.9; #Dissociation constant for olfactory bulb (/hr)

KDCB = 99.9; #Dissociation constant for cerebellum (/hr)

KDPT = 99.9; #Dissociation constant for pituitary (/hr)

KAST = 99.9; #Association constant for striatum (/ug/L/hr)

KAOB = 99.9; #Association constant for olfactory bulb (/ug/L/hr)

KACB = 99.9; #Association constant for cerebellum (/ug/L/hr)

KAPT = 99.9; #Association constant for pituitary (/ug/L/hr)

# Maximal storage tissue capacities (fitted with data, ug/L tissue)

BMAXLIVC = 99.9; #liver

BMAXBODC = 99.9; #others

BMAXBONEC = 99.9; #bone

BMAXLUNGC = 99.9; #lung

BMAXSTC = 99.9; #striatum

BMAXOBC = 99.9; #olfactory bulb

BMAXCBC = 99.9; #cerebellum

BMAXPTC = 99.9; #pituitary

# Dose-dependant induction constants (fitted with data)

# Biliary induction

KBINDUC = 99.9; # maximal biliary induction factor (0 off or x times added)

KM = 99.9; # biliary affinity rate constant

SLOPE = 99.9; # slope factor induction

#********************************

# Dosing Controls

#********************************

# Dietary inputs

DDIET = 0.; # Diet (ppm)

BDIET = 0.; # Diet (mg/day)

FDIETUP = 99.9; # Diet absorption fraction

INFAC = 99.9; # Dietary Intake Factors (kg/day/BW) (from EPA 1986)

XDOFF = 99.9; # switch to turn diet off at day X

XDIN = 0.0; # Diet on by default

# Inhalation inputs

DINH = 0.; # Inhaled dose (mg/m3)

# IV tracer (54Mn)

DIVR = 0.; # IV tracer injection dose (uCi)

SA = 99.9; # Specific activity (uCi/ug)

SANEWLAND = 99.9; # Adjustment factor(?) for Tracer in feces

IVRON = 99.9; # first day of IV tracer (day)

LENDOSER = 0.0; # Length of IV (hrs)

FBQ = 37.; # Conversion Factor (kBq/uCi)

### These need to be forcing function

KORAL = 0.; # not used

KIVR = 0.; # iv 54Mn input rate (ug/hr)

KIPR = 0.; # radio-label dose input IP

IPFRAC = 0.;# Fraction of IP dose into IP comparment(rest straight to liver)

KIPSLOW = 0.;# Rate from IP compartment to Liver

DDIETR = 0.; # dose (uCi)

KFECES = 0.; # fecal excretion rate

KGI = 99.9; # Absorption rate of Mn from GI lumen to epithelium

FENT = 99.9; # Fraction Mn available to be absorbed from GI lumen

KENT = 99.9; # Rate of release from GI epithelium to lumen

## subcutaneous tracer (54Mn) **** new addition 07/14/09 ****

DINHR = 0.; # radio-label inhalation

FDEPX = 1.; # not used

DSUBQ = 0.; # radio-label subq dose

KSBQ = 0.; # absorption rate from subq injection site

KSQR = 0.0; # not used

SUBQ = 0.; # subcutaneous injection mixed hot/cold Mn (ug)

DIPR = 0.0; # Radio-labeled IP injection dose (ug/kg)

IPRON = 0.0; # first day of IP tracer (days)

#Initial amounts for state variables

#In order to have model start at steady state with background diet and/or inhalation

#state variables must be initialized to the steady state amount or the model must be run

#to steady state before assessing any additional exposure using DSTART which is the days to

#run the model prior to starting the study based exposure

#--------------------------------------------------------------------

# Placeholder declaration for scaled/combined variables calculated in

# "Initialize" section

#--------------------------------------------------------------------

#These variables have values assigned in the "Initialize" section, but they must be declared here (as global variables) so that they can be used in the Dynamics section.

#Therefore they will be declared here with placeholder values of 0.

ADEPLU0 = 0 ;

ALUBD0 = 0 ;

ALUNG0 = 0 ;

BMNLUNG0 = 0 ;

ADEPNR0 = 0 ;

ANOSE0 = 0 ;

ANOLF0 = 0 ;

ABILE0 = 0 ;

ALBD0 = 0 ;

AL0 = 0 ;

BMNLIV0 = 0 ;

AGL0 = 0 ;

AGE0 = 0 ;

FECES0 = 0 ;

ALGL0 = 0 ;

ABRBD0 = 0 ;

ABRST0 = 0 ;

BMNST0 = 0 ;

ABROB0 = 0 ;

BMNOB0 = 0 ;

ABRCB0 = 0 ;

BMNCB0 = 0 ;

ABRPT0 = 0 ;

BMNPT0 = 0 ;

ABOBD0 = 0 ;

ABONE0 = 0 ;

BMNBONE0 = 0 ;

AOBD0 = 0 ;

AO0 = 0 ;

BMNOTH0 = 0 ;

AART0 = 0 ;

AVEN0 = 0 ;

ATOT0 = 0; #initial total body burden

#END #Initial

Initialize{

#**********************************************

# Initial/background tissue Mn amounts

#**********************************************

#Set initial values of state variables to the appropriate input parameters

#Free Mn amounts (ug)

ADEPLU = ADEPLU0 ;

ALUBD = ALUBD0 ;

ALUNG = ALUNG0 ;

BMNLUNG = BMNLUNG0 ;

ADEPNR = ADEPNR0 ;

ANOSE = ANOSE0 ;

ANOLF = ANOLF0 ;

ABILE = ABILE0 ;

ALBD = ALBD0 ;

AL = AL0 ;

BMNLIV = BMNLIV0 ;

AGL = AGL0 ;

AGE = AGE0 ;

FECES = FECES0 ;

ALGL = ALGL0 ;

ABRBD = ABRBD0 ;

ABRST = ABRST0 ;

BMNST = BMNST0 ;

ABROB = ABROB0 ;

BMNOB = BMNOB0 ;

ABRCB = ABRCB0 ;

BMNCB = BMNCB0 ;

ABRPT = ABRPT0 ;

BMNPT = BMNPT0 ;

ABOBD = ABOBD0 ;

ABONE = ABONE0 ;

BMNBONE = BMNBONE0 ;

AOBD = AOBD0 ;

AO = AO0 ;

BMNOTH = BMNOTH0 ;

AART = AART0 ;

AVEN = AVEN0 ;

#Save initial total body burden in a separate variable, for use later

ATOT0 = ADEPLU0 + ALUBD0 + ALUNG0 + BMNLUNG0 + ADEPNR0 + ANOSE0 + ANOLF0 + ABILE0 + ALBD0 + AL0 + BMNLIV0

+ AGL0 + AGE0 + FECES0 + ALGL0 + ABRBD0 + ABRST0 + BMNST0 + ABROB0 + BMNOB0 + ABRCB0 + BMNCB0 + ABRPT0

+ BMNPT0 + ABOBD0 + ABONE0 + BMNBONE0 + AOBD0 + AO0 + BMNOTH0 + AART0 + AVEN0;

atotbody = ATOT0;

} #End of Initialize section

#*******************************************************************************

#*******************************************************************************

#*******************************************************************************

Dynamics{

#************************************

# Scaling calculations

#************************************

# Tissue volumes (L)

VBLD = VBLDC * BW; # whole blood (arterial/venous)

VBONE = VBONEC * BW; # bone

VLIV = VLIVC * BW; # liver

VLUNG = VLUNGC * BW; # lung

VNOLF = SANOLF * pow(BW,0.75) * THNOSE / 10000. / 1000.; # nasal olfactory region

VNRESP = SANRESP * pow(BW,0.75) * THNOSE / 10000. / 1000.; # nasal respiratory region

VNOSE = VNOLF + VNRESP; # nasal cavity total

VBRN = VBRNC * BW; # whole brain

VSTM = VSTMC * VBRN; # striatum (globus pallidus for monkey and human)

VOFB = VOFBC * VBRN; # olfactory bulb

VCBM = VCBMC * VBRN; # cerebellum

VPIT = VPITC * VBRN; # pituitary (monkey and human only)

VOTH = BW-VSTM-VOFB-VCBM-VLIV-VBONE-VLUNG-VBLD-VNOSE-VPIT; # Other tissues

# Tissue blood flows (L/hr)

QC = QCC*pow(BW,0.75); # cardiac Output

QP = QPC*pow(BW,0.75); # alveolar ventilation

QLIV = QLIVC*QC; # liver

QBRN = QBRNC*QC*((VSTMC+VOFBC+VCBMC+VPITC)/VBRNC); # brain tissues simulated

QBONE = QBONEC*QC; # bone

QNOSE = QNOSEC*QC; # nose

QOTH = QC-QBONE-QBRN-QLIV-QNOSE; # other tissues

QBILE = QBILEC*BW ; #Bile flow rate for monkey (3.5 ml/hr in a 4.5 kg monkey from Beaudoin et al. 1975)

# Scale biliary excretion to BW^0.75

KBILE = KBILEC * pow(BW,0.75); #Biliary elimination (L/hr)

# Scale nasal and lung epithelium clearances per BW^0.25

KDEPLU = KDEPLUC*pow(BW,-0.25); #Deposited lung clearance to deep tissues (/hr)

KSHALLU = KSHALLUC*pow(BW,-0.25); #Deposited lung clearance to deep tissues (/hr)

KDEPNR = KDEPNRC*pow(BW,-0.25); #Deposited nose clearance to blood (/hr)

KNPOB = KNPOBC*pow(BW,-0.25); #Nose clearance to brain (/hr)

###############################################################################

# Scale influx/efflux rates to BW^0.25

KINST = KINSTC * pow(BW,-0.25); # striatum influx (/hr)

KINOB = KINOBC * pow(BW,-0.25); # olfactory bulb influx (/hr)

KINCB = KINCBC * pow(BW,-0.25); # cerebellum influx (/hr)

KINPT = KINPTC * pow(BW,-0.25); # pituitary influx (/hr)

KINLUNG = KINLUNGC * pow(BW,-0.25); # Lung influx rate constant

KINLIV = KINLIVC * pow(BW,-0.25); # Liver influx rate constant

KINBONE = KINBONEC * pow(BW,-0.25); # Bone influx rate constant

KINOTH = KINOTHC * pow(BW,-0.25); # Rest of body (Other) influx rate constant

# First order rate * Tissue vol = clearance (/hr)

KOUTST = KOUTSTC*pow(BW,-0.25) ; # striatum efflux (/hr)

KOUTOB = KOUTOBC*pow(BW,-0.25) ; # olfactory bulb efflux (/hr)

KOUTCB = KOUTCBC*pow(BW,-0.25) ; # cerebellum efflux (/hr)

KOUTPT = KOUTPTC*pow(BW,-0.25) ; # pituitary efflux (/hr)

KOUTLUNG = KOUTLUNGC*pow(BW,-0.25) ; # Lung efflux rate constant (/hr)

KOUTLIV = KOUTLIVC*pow(BW,-0.25) ; # Liver efflux rate constant (/hr)

KOUTBONE = KOUTBONEC*pow(BW,-0.25) ; # Bone efflux rate constant (/hr)

KOUTOTH = KOUTOTHC*pow(BW,-0.25) ; # Rest of body (Other) efflux rate constant (/hr)

###############################################################################

# Tissue maximal capacities

BMAXLIV = BMAXLIVC * VLIV; #liver

BMAXBOD = BMAXBODC * VOTH; #others

BMAXBONE = BMAXBONEC * VBONE; #bone

BMAXLUNG = BMAXLUNGC * VLUNG; #lung

BMAXST = BMAXSTC * VSTM; #striatum

BMAXOB = BMAXOBC * VOFB; #olfactory bulb

BMAXCB = BMAXCBC * VCBM; #cerebellum

BMAXPT = BMAXPTC * VPIT; #pituitary

#***************************************

# Binding Statements

#***************************************

#BLung = binding capacity in LUNG(ug)

BLUNG = BMAXLUNG - BMNLUNG - XBMNLUNG; # Free tissue capacity (ug)

PCBLUNG = BLUNG / BMAXLUNG * 100; # % Free capacity

#Liver tissue binding capacity

BLIV = BMAXLIV - BMNLIV - XBMNLIV; # Free tissue capacity (ug)

PCBLIV = BLIV / BMAXLIV * 100.; # % Free capacity

#Mn binding capacity in STM (ug)

BST = BMAXST - BMNST - XBMNST; # Free STM capacity (ug)

PCBST = BST / BMAXST * 100.; # % Free capacity

#Mn binding capacity in Olfactory bulb (ug)

BOB = BMAXOB - BMNOB - XBMNOB; # Free STM capacity (ug)

PCBOB = BOB / BMAXOB * 100.; # % Free capacity

#Mn binding capacity in CBM (ug)

BCB = BMAXCB - BMNCB - XBMNCB; # Free CBM capacity (ug)

PCBCB = BCB / BMAXCB * 100.; # % Free capacity

#Mn binding capacity in PIT (ug)

BPT = BMAXPT - BMNPT - XBMNPT; # Free PIT capacity (ug)

PCBPT = BPT / BMAXPT * 100.; # % Free capacity

#Bbone = binding Capacity BONE(ug)

BBONE = BMAXBONE - BMNBONE - XBMNBONE; # Free tissue capacity (ug)

PCBBONE = BBONE / BMAXBONE * 100.; # % Free capacity

#Other tissue binding capacity

BOTH = BMAXBOD - BMNOTH - XBMNOTH; # Free tissue capacity (ug)

PCBOTH = BOTH / BMAXBOD * 100; # % Free capacity

#********************************

# Dosing calculations

#********************************

# # Mn Diet uptake

KDIET = (BDIET+(DDIET*INFAC*pow(BW,0.75)))*1000/24; #Dose mg/day or ppm to ug/hr

#***************************************

# Concentration Definitions

#***************************************

#Manganese

CVLUNG = ALUBD/(VLUNG*0.03); # Venous lung blood Mn concentration (ug/kg)

CLUNG = ALUNG/VLUNG; # Lung Mn concentration (free, ug/kg)

CVNOSE = ANOSE/(VNRESP*0.03); # Nasal Respiratory Venous Mn concentration (ug/kg)

CVL = ALBD/(VLIV*0.03); # Lung Venous Mn concentration (ug/kg)

CLIV = AL/VLIV; # Liver Venous Mn concentration (ug/kg)

CVBR = ABRBD/(VBRN*0.03); # Brain Venous Mn concentration for the four regions of the brain (ug/kg)

CBRST = ABRST / VSTM; # Free STM Mn concentration (ug/kg)

CBMNST = BMNST / VSTM; # Bound STM Mn concentration (ug/kg)

CBROB = ABROB / VOFB; # Free OFB Mn concentration (ug/kg)

CBRCB = ABRCB / VCBM; # Free CBM Mn concentration (ug/kg)

CBRPT = ABRPT / VPIT; # Free PIT Mn concentration (ug/kg)

CVBONE = ABOBD /(VBONE*0.03); # based on blood volume in the bone (ug/kg) # Free Mn concentration leaving the bone (ug/kg)

CBONE = ABONE/VBONE;

CVO = AOBD /(VOTH*0.03); # based on blood volume in the brain (ug/kg) # Free Mn concentration leaving the brain (ug/kg)

COTH = AO/VOTH;

CA = AART/VBLD; # Arteril blood conc. (ug/kg)

CART = AART/VBLD/1000.; # Areterial blood conc. (ug /g)

CV = AVEN/VBLD; # Venous blood conc. (ug/kg)

#Tracer Manganese (same as above with X to denote tracer)

XCVLUNG = XALUBD/(VLUNG*0.03);

XCLUNG = XALUNG/VLUNG;

XCVNOSE = XANOSE/(VNRESP*0.03);

XCVL = XALBD /(VLIV*0.03);

XCLIV = XAL/VLIV;

XCVBR = XABRBD/(VBRN*0.03);

XCBRST = XABRST/VSTM;

XCBROB = XABROB/VOFB;

XCBRCB = XABRCB/VCBM;

XCBRPT = XABRPT/VPIT;

XCVBONE = XABOBD /(VBONE*0.03);

XCBONE = XABONE/VBONE;

XCVO = XAOBD /(VOTH*0.03);

XCOTH = XAO/VOTH;

XCA = XAART/VBLD;

XCART = XAART/VBLD/1000;

XCV = XAVEN/VBLD;

#***************************************

# Model differential Equations

#***************************************

##############################################################################

# ******* LUNG *******

##############################################################################

# Adeplu = amount deposited into lungs (ug)

KINHLU = QP*DINH*FDEPLU*EXPOSURE ;

RADEPLU = KINHLU - KDEPLU*ADEPLU - KSHALLU*ADEPLU ; # Rate of Mn on lung epithelium over time (ug/hr)

dt(ADEPLU) = RADEPLU ; # Amount of Mn on lung epithelium (ug)

# Tissue blood in Lung

##############################################################################

RALUBD = QC*(CV-CVLUNG) + KOUTLUNG*ALUNG - KINLUNG*ALUBD ;

dt(ALUBD) = RALUBD ; # Free Mn in lung blood (ug)

RALUNG = KINLUNG*ALUBD - KOUTLUNG*ALUNG + KDLUNG*BMNLUNG - KALUNG*BLUNG*CLUNG + KDEPLU*ADEPLU ;

dt(ALUNG) = RALUNG ;

RBMNLUNG = -KDLUNG*BMNLUNG + KALUNG*BLUNG*CLUNG ;

dt(BMNLUNG) = RBMNLUNG ;

##############################################################################

# ******* NOSE *******

##############################################################################

KINHNR = QP*DINH*FDEPNR*EXPOSURE ;

# Deposited nasal respiratory

RADEPNR = KINHNR - KDEPNR*ADEPNR ; # Mn on nasal cavity over time (ug/hr)

dt(ADEPNR) = RADEPNR ; # Mn on nasal cavity (ug)

# Nasal respiratory blood

RANOSE = QNOSE*(CA - CVNOSE) + KDEPNR*ADEPNR ; # Mn on respiratory epithelium over time (ug/hr)

dt(ANOSE) = RANOSE ; # Mn on respiratory epithelium (ug)

# Deposited olfactory region

KINHNO = QP*DINH*FDEPNO*EXPOSURE ;

RANOLF = KINHNO - KNPOB*ANOLF ; # Mn on ofactory epithelium over time (ug/hr)

dt(ANOLF) = RANOLF ; # Mn on ofactory epithelium (ug)

##############################################################################

# ******* LIVER *******

##############################################################################

# Bile Mn concentration (induction)

KBILEINC = (1 + KBINDUC*pow((CART+XCART),SLOPE)/(pow(KM,SLOPE)+pow((CART+XCART),SLOPE))) ;

KBILEX = KBILE*(1 + (KBINDUC*pow((CART+XCART),SLOPE))/(pow(KM,SLOPE)+pow((CART+XCART),SLOPE))); # free Mn dependent excretion rate constant (L/hr)

#*******3/7/2013. (CLIV is free Mn conc in liver for biliary transport)

RBILE = KBILEX*CLIV ; # biliary excretion rate (ug/hr)

CBILE = RBILE/QBILE ; # concentration in bile excreted per time (ug/L)

RLOSS = QBILE*CBILE ; # bile flow elimination

RABILE = RBILE - RLOSS ; # amount of Mn in bile over time (ug/hr)

dt(ABILE) = RABILE ; # amount of Mn in bile (ug)

# tissue blood in Liver

RALBD = QLIV*(CA-CVL) + KOUTLIV*AL - KINLIV*ALBD ; # Free Mn in liver blood over time (ug/hr)

dt(ALBD) = RALBD; # Free Mn in liver blood (ug)

RADI = (KDIET*XDIN + RBILE)*FDIETUP; # RATE OF DIETARY MN INPUT TO LIVER

RAL = KINLIV*ALBD - KOUTLIV*AL + KDLIV*BMNLIV - KALIV*BLIV*CLIV - RBILE + RADI ;

dt(AL) = RAL;

RBMNLIV = -KDLIV*BMNLIV + KALIV*BLIV*CLIV;

dt(BMNLIV) = RBMNLIV;

##GUT

##############################################################################

RAGL = (KDIET*XDIN)*(1-FDIETUP) - (1-FENT)*KGI*AGL - FENT*KGI*AGL ; ## RATE OF CHANGE IN GUT LUMEN

dt(AGL) = RAGL; ## AMOUNT OF MN IN GUT LUMEN (NOT AVAILABLE TO SYSTEMIC, NOT UPTAKEN)

RAGE = FENT*KGI*AGL - KENT*AGE; ## RATE OF MN IN GI EPITHELIUM

dt(AGE) = RAGE; ## AMOUNT OF MN IN ENTEROCYTES

RFECES = KFECES*ALGL; ## UG MN/HR

dt(FECES) = RFECES; ## AMOUNT IN FECES

RALGL = (1-FENT)*KGI*AGL - RFECES + KENT*AGE + RBILE*(1-FDIETUP) ;

dt(ALGL) = RALGL; ## AMOUNT OF MN IN LOWER GI

##############################################################################

# ******* BRAIN *******

##############################################################################

#Free Mn in brain blood supply

RABRBD = QBRN*(CA - CVBR) + KOUTST*ABRST + KOUTOB*ABROB + KOUTCB*ABRCB + KOUTPT*ABRPT - (KINST + KINOB + KINCB + KINPT)*ABRBD; # Free Mn in brain blood over time (ug/hr)

dt(ABRBD) = RABRBD; # Free Mn in brain blood (ug)

##############################################################################

#Free Mn in brain tissue (ug) - STRIATUM

##############################################################################

RABRST = KINST*ABRBD - KOUTST*ABRST + KDST*BMNST - KAST*BST*CBRST; # Free Mn in STM over time (ug/hr)

dt(ABRST) = RABRST; # Free Mn in STM (ug)

#Bound Mn in brain tissue (ug) - STRIATUM

RBMNST = - KDST*BMNST + KAST*BST*CBRST; # Mn stored in STM over time (ug/hr)

dt(BMNST) = RBMNST; # Mn stored in STM (ug)

##############################################################################

#Free Mn in brain tissue (ug) - OLFACTORY BULB

##############################################################################

RABROB = KINOB*ABRBD - KOUTOB*ABROB + KDOB*BMNOB - KAOB*BOB*CBROB + KNPOB*ANOLF ;

dt(ABROB) = RABROB; # Free Mn in OFB (ug)

#Bound Mn in brain tissue (ug) - OLFACTORY BULB

RBMNOB = - KDOB*BMNOB + KAOB*BOB*CBROB; # Mn stored in OFB over time (ug/hr)

dt(BMNOB) = RBMNOB; # Mn stored in OFB (ug)

##############################################################################

#Free Mn in brain tissue (ug) - CEREBELLUM

##############################################################################

RABRCB = KINCB*ABRBD - KOUTCB*ABRCB + KDCB*BMNCB - KACB*BCB*CBRCB;

dt(ABRCB) = RABRCB; # Free Mn in CBM (ug)

#Bound Mn in brain tissue (ug) - CEREBELLUM

RBMNCB = - KDCB*BMNCB + KACB*BCB*CBRCB; # Mn stored in CBM over time (ug/hr)

dt(BMNCB) = RBMNCB; # Mn stored in CBM (ug)

##############################################################################

# PITUITARY

#Free Mn in brain tissue (ug) - PITUITARY

##############################################################################

RABRPT = KINPT*ABRBD - KOUTPT*ABRPT + KDPT*BMNPT - KAPT*BPT*CBRPT; # Free Mn in PIT over time (ug/hr)

dt(ABRPT) = RABRPT; # Free Mn in PIT (ug)

#Bound Mn in brain tissue (ug) - PITUITARY

RBMNPT = - KDPT*BMNPT + KAPT*BPT*CBRPT; # Mn stored in PIT over time (ug/hr)

dt(BMNPT) = RBMNPT; # Mn stored in PIT (ug)

##############################################################################

# ******* BONE *******

##############################################################################

RABOBD = QBONE*(CA - CVBONE) + KOUTBONE*ABONE - KINBONE * ABOBD; # Free Mn in bone blood over time (ug/hr)

dt(ABOBD) = RABOBD; # Free Mn in bone blood (ug)

RABONE = KINBONE*ABOBD - KOUTBONE*ABONE + KDBONE*BMNBONE - KABONE*BBONE*CBONE;

dt(ABONE) = RABONE;

RBMNBONE = - KDBONE*BMNBONE + KABONE*BBONE*CBONE; # Mn stored in tissue over time (ug/hr)

dt(BMNBONE) = RBMNBONE; # Mn stored in tissue (ug)

# ******* REST OF BODY (OTHERS) *******

##############################################################################

RAOBD = QOTH*(CA - CVO) + KOUTOTH*AO - KINOTH * AOBD; # Free Mn in brain blood over time (ug/hr)

dt(AOBD) = RAOBD; # Free Mn in brain blood (ug)

RAO = KINOTH*AOBD - KOUTOTH*AO + KDOTH*BMNOTH - KAOTH*BOTH*COTH;

dt(AO) = RAO;

RBMNOTH = - KDOTH * BMNOTH + KAOTH*BOTH*COTH; # Mn stored in tissue over time (ug/hr)

dt(BMNOTH) = RBMNOTH; # Mn stored in tissue (ug)

#BLOOD

#Free Mn arterial blood concentration (ug)

##############################################################################

RAART = QC*CVLUNG - QC*CA + KSHALLU*ADEPLU ;

dt(AART) = RAART;

#Free Mn venous blood concentration (ug)

##############################################################################

RAVEN = QLIV*CVL + QBRN*CVBR + QOTH*CVO + QBONE*CVBONE + QNOSE*CVNOSE - QC*CV + KSBQ*ASQ ;

dt(AVEN) = RAVEN;

## Subcutaneous exposure

RASQ = KSUBQ - KSBQ*ASQ;

dt(ASQ) = RASQ;

#***********************************************************************

#***********************************************************************

# Tracer Kinetics

#***********************************************************************

#***********************************************************************

XRADEPLU = QP*KINHR - KDEPLU*XADEPLU - KSHALLU*XADEPLU;

dt(XADEPLU) = XRADEPLU;

#LUNG

XRALUBD = QC*(XCV-XCVLUNG) + KOUTLUNG*XALUNG - KINLUNG*XALUBD ;

dt(XALUBD) = XRALUBD; # Free Mn in brain blood (ug)

XRALUNG = KINLUNG*XALUBD - KOUTLUNG*XALUNG + KDLUNG*XBMNLUNG - KALUNG*BLUNG*XCLUNG + KDEPLU*XADEPLU ;

dt(XALUNG) = XRALUNG;

XRBMNLUNG = -KDLUNG*XBMNLUNG + KALUNG*BLUNG*XCLUNG;

dt(XBMNLUNG) = XRBMNLUNG;

# Nasal respiratory blood

XRANOSE = QNOSE*(XCA - XCVNOSE) ;

dt(XANOSE) = XRANOSE;

##############################################################################

##############################################################################

##############################################################################

#LIVER

XRAIP = KIPR*IPFRAC - KIPSLOW*XAIP ;# Peritoneal Space

dt(XAIP) = XRAIP;

XRALBD = QLIV*(XCA-XCVL) + KOUTLIV*XAL - KINLIV*XALBD; # Free Mn in brain blood over time (ug/hr)

dt(XALBD) = XRALBD; # Free Mn in brain blood (ug)

XRBILE = KBILEX*XCLIV;

XRADI = (KDIETR*XDIN + XRBILE)*FDIETUP; ## RATE OF DIETARY MN INPUT TO LIVER (ug/hr)

XRAL = KINLIV*XALBD - KOUTLIV*XAL + KDLIV*XBMNLIV - KALIV*BLIV*XCLIV - XRBILE + XRADI + KIPR*(1-IPFRAC) + KIPSLOW*XAIP;

dt(XAL) = XRAL;

XRBMNLIV = -KDLIV*XBMNLIV+KALIV*BLIV*XCLIV;

dt(XBMNLIV) = XRBMNLIV;

##############################################################################

##############################################################################

##############################################################################

#Gut

XRAGL = (KDIETR*XDIN)*(1-FDIETUP) - (1-FENT)*KGI*XAGL - FENT*KGI*XAGL ; ## RATE OF CHANGE IN GUT LUMEN

dt(XAGL) = XRAGL; ## AMOUNT OF MN IN GUT LUMEN (NOT AVAILABLE TO SYSTEMIC, NOT UPTAKEN)

XRAGE = FENT*KGI*XAGL - KENT*XAGE; ## RATE OF MN IN GI EPITHELIUM

dt(XAGE) = XRAGE; ## AMOUNT OF MN IN ENTEROCYTES

XRFECES = KFECES*XALGL;

XRALGL = (1-FENT)*KGI*XAGL - XRFECES + KENT*XAGE + XRBILE*(1-FDIETUP) ;

dt(XALGL) = XRALGL; ## AMOUNT OF MN IN LOWER GI

dt(XFECES) = XRFECES; ## AMOUNT IN FECES

##############################################################################

##############################################################################

##############################################################################

#BRAIN

##############################################################################

#ABrBd = Amount free in brain blood (ug)

XRABRBD = QBRN*(XCA - XCVBR) + KOUTST*XABRST + KOUTOB*XABROB + KOUTCB*XABRCB + KOUTPT*XABRPT

- (KINST + KINOB + KINCB + KINPT)*XABRBD;

dt(XABRBD) = XRABRBD;

#ABrST = Amount free in brain tissue (ug) - STRIATUM

##############################################################################

XRABRST = KINST*XABRBD - KOUTST*XABRST + KDST*XBMNST - KAST*BST*XCBRST;

dt(XABRST) = XRABRST;

#BMnST = Amount bound in brain tissue (ug) - STRIATUM

XRBMNST = - KDST*XBMNST + KAST*BST*XCBRST;

dt(XBMNST) = XRBMNST;

#ABrOB = Amount free in brain tissue (ug) - OLFACTORY BULB

##############################################################################

XRABROB = KINOB*XABRBD - KOUTOB*XABROB + KDOB*XBMNOB - KAOB*BOB*XCBROB;

dt(XABROB) = XRABROB;

#BMnOB = Amount bound in brain tissue (ug) - OLFACTORY BULB

XRBMNOB = - KDOB*XBMNOB + KAOB*BOB*XCBROB;

dt(XBMNOB) = XRBMNOB;

#ABrCB = Amount free in brain tissue (ug) - CEREBELLUM

##############################################################################

XRABRCB = KINCB*XABRBD - KOUTCB*XABRCB + KDCB*XBMNCB - KACB*BCB*XCBRCB;

dt(XABRCB) = XRABRCB;

#BMnCB = Amount bound in brain tissue (ug) - CEREBELLUM

XRBMNCB = - KDCB*XBMNCB + KACB*BCB*XCBRCB;

dt(XBMNCB) = XRBMNCB;

#ABrPT = Amount free in brain tissue (ug) - PITUITARY

##############################################################################

XRABRPT = KINPT*XABRBD - KOUTPT*XABRPT + KDPT*XBMNPT - KAPT*BPT*XCBRPT;

dt(XABRPT) = XRABRPT;

#BMnPT = Amount bound in brain tissue (ug) - PITUITARY

XRBMNPT = - KDPT*XBMNPT + KAPT*BPT*XCBRPT;

dt(XBMNPT) = XRBMNPT;

##############################################################################

##############################################################################

##############################################################################

#BONE

##############################################################################

XRABOBD = QBONE*(XCA - XCVBONE) + KOUTBONE*XABONE - KINBONE*XABOBD; # Free Mn in brain blood over time (ug/hr)

dt(XABOBD) = XRABOBD; # Free Mn in brain blood (ug)

XRABONE = KINBONE*XABOBD - KOUTBONE*XABONE +KDBONE*XBMNBONE - KABONE*BBONE*XCBONE;

dt(XABONE) = XRABONE;

XRBMNBONE = - KDBONE*XBMNBONE + KABONE*BBONE*XCBONE; # Mn stored in tissue over time (ug/hr)

dt(XBMNBONE) = XRBMNBONE; # Mn stored in tissue (ug)

#############################################################################

#############################################################################

#############################################################################

#REST OF BODY

#############################################################################

XRAOBD = QOTH*(XCA - XCVO) + KOUTOTH*XAO - KINOTH*XAOBD; # Free Mn in brain blood over time (ug/hr)

dt(XAOBD) = XRAOBD; # Free Mn in brain blood (ug)

XRAO = KINOTH*XAOBD - KOUTOTH*XAO + KDOTH*XBMNOTH - KAOTH*BOTH*XCOTH;

dt(XAO) = XRAO;

XRBMNOTH = - KDOTH*XBMNOTH + KAOTH*BOTH*XCOTH; # Mn stored in tissue over time (ug/hr)

dt(XBMNOTH) = XRBMNOTH; # Mn stored in tissue (ug)

############################################################################

############################################################################

############################################################################

#BLOOD

############################################################################

#CA = Arterial blood concentration (ug)

XRAART = QC*XCVLUNG - QC*XCA + KSHALLU*XADEPLU;

dt(XAART) = XRAART;

#CV = Venous blood concentration (ug)

XRAVEN = QLIV*XCVL + QBRN*XCVBR + QOTH*XCVO + QBONE*XCVBONE - QC*XCV + QNOSE*XCVNOSE + KSBQ*XASQ + KIVR ;

dt(XAVEN) = XRAVEN;

## Subcutaneous

XRASQ = KSUBQR - KSBQ*XASQ;

dt(XASQ) = XRASQ;

#############################################################################

#############################################################################

#******************************

# Mass balance (ug)

#******************************

dt(LOSS) = RFECES; # Amount loss in bile

RDOSE = KDIET + (KINHLU + KINHNO + KINHNR)*EXPOSURE; # Total intake rate

dt(DOSE) = RDOSE; # Total Dose

dt(DOSE54) = KIVR + KDIETR + KIPR + KSUBQR + QP*KINHR ; # Total Dose

dt(XLOSS) = XFECES; # loss in bile

};

#############################################################################

#############################################################################

CalcOutputs{

### Forcing function checks

xdin = XDIN;

dietary = KDIET*XDIN;

exposure = EXPOSURE;

inhalation = (KINHLU+KINHNO+KINHNR)*EXPOSURE;

kivr = KIVR;

intravenous = KIVR;

kdietr = KDIETR;

# Cold Mn mass

# AtotLung = Total amount of Mn in LUNG(ug)

atotlung = ALUNG + ALUBD + BMNLUNG + ADEPLU; # ug

ctotlung = atotlung/(VLUNG*1000); # ug /g tissue

atotnose = ANOSE + ADEPNR + ANOLF; # ug

ctotnose = atotnose/(VNOSE*1000); # ug /g tissue

atotliv = AL + ALBD+BMNLIV; # ug

ctotliv = atotliv/(VLIV*1000); # ug /g tissue

#Bile concentration

cbile = CBILE/1000 ; #ug/g

# total amount in STM

ast = ABRST + BMNST + ABRBD*VSTMC; # ug

ctotst = ast / VSTM /1000; # ug /g tissue

# total - OFB

aob = (ABROB + BMNOB) + ABRBD*VOFBC; # ug

ctotob = (aob) / VOFB /1000; # ug /g tissue

# total in CBM

acb = (ABRCB + BMNCB) + ABRBD*VCBMC; # ug

ctotcb = (acb)/VCBM/1000; # ug /g tissue

# total in PIT

apt = (ABRPT + BMNPT) + ABRBD*VPITC ; # ug

ctotpt = (apt)/VPIT/1000; # ug /g tissue

# AtotBr = Total amount of Mn in Brain tissues(ug)

abr = ABRST + ABROB + ABRCB + ABRPT; # Free Mn in brain regions (ug/hr)

bmnbr = BMNST + BMNOB + BMNCB + BMNPT; # Mn stored in brain regions (ug)

cprotmnbr = bmnbr/(VSTM+VOFB+VCBM+VPIT); # Bound brain region Mn concentration (ug/kg)

atotbr = abr + bmnbr + ABRBD; # Total brain Mn incl. blood (ug)

ctotbr = atotbr/((VSTM+VOFB+VCBM+VPIT+VBRN*0.03)*1000); # ug /g tissue

atotbone = ABONE + ABOBD + BMNBONE;

ctotbone = atotbone/(VBONE*1000); # ug /g tissue

atototh = AO + AOBD + BMNOTH ;

ctototh = atototh/(VOTH*1000) ; # ug /g tissue

atotgut = AGL + AGE + ALGL ;

atotbody = atotliv + atotbr + atototh + AART + AVEN + atotbone + atotlung + atotnose + atotgut; # Body burden

mass = atotbody - ATOT0 ; # Tissue Mass from intake

xatotlung = XALUNG + XALUBD+XBMNLUNG + XADEPLU; # ug

xctotlung = xatotlung/(VLUNG*1000);

xatotliv = XAL + XALBD+XBMNLIV; # ug

xctotliv = xatotliv/(VLIV*1000);

# total amount in STM

xast = XABRST + XBMNST + XABRBD*VSTMC;

# total - OFB

xaob = (XABROB + XBMNOB) + XABRBD*VOFBC;

# total in CBM

xacb = (XABRCB + XBMNCB) + XABRBD*VCBMC;

# total in PIT

xapt = (XABRPT + XBMNPT) + XABRBD*VPITC ;

# AtotBr = Total amount of Mn in Brain tissues(ug)

xabr = XABRST + XABROB + XABRCB + XABRPT;

xbmnbr = XBMNST + XBMNOB + XBMNCB + XBMNPT;

xatotbr = xabr + XABRBD + xbmnbr;

xatotbone = XABONE + XABOBD +XBMNBONE;

xctotbone = xatotbone/(VBONE*1000);

xatototh = XAO + XAOBD +XBMNOTH;

xctototh = xatototh/(VOTH*1000); # ug /g tissue

xatotgut = XAGL + XAGE + XALGL;

# liver, brain, other, blood (art, ven), bone, lung, nose, gut, IP

atot54 = xatotliv + xatotbr + xatototh + XAART + XAVEN + xatotbone + xatotlung + XANOSE + xatotgut + XAIP; # Tracer Body burden (ug)

# Whole body tracer activity (kBq) -- used to compared with data

atotmn54 = atot54 * SA * FBQ;

#Point Conc (mg Mn/g feces), assuming 12g/day fecal volume)

xrfecesmgtotal = SANEWLAND*XRFECES/1000/0.5; #0.5 - 12 g/day

qbalance = QC - QOTH - QBRN - QLIV - QNOSE - QBONE;

vbalance = BW - VSTM - VOFB - VCBM - VLIV - VBONE - VLUNG - VBLD - VNOSE - VPIT - VOTH ;

balance = (DOSE > 0.0 ? ((mass + LOSS)/DOSE) : 0.0); # Value close to 1 means the model is mass balanced for Mn

balance54 = (DOSE54 > 0.0 ? (atot54 + XLOSS)/(DOSE54) : 0.0); # Value close to 1 means the model is mass balanced for 54Mn

ret54 = (DOSE54 > 0.0 ? (atot54 / DOSE54)*100 : 0.0);

};

End. # END OF MODEL

############################################################################

############################################################################
